# A qualitative metasynthesis of stigma in women living with HIV in the United States

**DOI:** 10.1186/s12939-023-01969-5

**Published:** 2023-08-21

**Authors:** Sadie Sommer, Julie Barroso

**Affiliations:** School of Nursing, Vanderbilt University, 461 21st Ave, S, Nashville, TN 37240 USA

**Keywords:** Stigma, Qualitative metasynthesis, HIV, Women, U.S

## Abstract

Our goal was to synthesize qualitative studies on HIV-related stigma as experienced by women living with HIV (WLWH) in the U.S. Qualitative metasynthesis techniques as developed by Sandelowski et al. (Res Nurs Health 30(1):99–111, 2007) were used to integrate and update findings on stigma in WLWH in the U.S. in 43 reports of qualitative studies conducted between 2004 and 2023 with 1118 participants. Developed themes explored a collective narrative of women first surviving the intersectionality of multiple sources of stigma, discovering non-linear pathways to transcend their stigma, and finally experiencing resilience through their transcendence of stigma. While this metasynthesis revealed similarities to an earlier metasynthesis in the ubiquity and persistence of stigma, they differ primarily in women’s abilities to find agency in managing and opposing their stigma. This cognitive reframing of their stigma helped women to redefine stigma as ignorance and move towards a more positive assessment of the self. In doing so, they separated themselves from their stigma and the damaging effects of it. Findings from this metasynthesis may serve as a useful tool for the development of stigma reduction interventions specific to the needs and experiences of WLWH in the U.S.

**Supplementary Information:**

The online version contains supplementary material available at 10.1186/s12939-023-01969-5.

## Introduction

More than 40 years after the first reported cases of AIDS, stigma remains a persistent problem for those who are living with HIV and impacts every step of the HIV care continuum, particularly engagement and retention in care [1, 2]. Stigma is widely seen as a barrier to ending the epidemic [3]. The psychosocial effects of HIV-related stigma are profound and can impact every aspect of an individual’s health and well-being [4]. The intersectional nature of stigma as it is experienced in HIV infection is compounded by gender, with women being impacted more adversely than men [5], largely due to their reproductive capabilities and the mistaken belief that women must have been infected by drug use or sex work [6].

The first qualitative metasynthesis on stigma in women living with HIV (WLWH) was conducted in 2004 as part of an NINR-funded project to develop the processes for conducting metasyntheses [7] Much has changed in the landscape of HIV in the succeeding 19 years; however, reducing HIV-related stigma remains an essential aspect of ending the HIV epidemic in the United States [8] and a current priority for the National Institutes of Health [1]. Understanding the progression in qualitative research and the current state of stigma for WLWH in the U.S. critically assists in informing on advancements in HIV-related stigma and pathways forwards. Therefore, we aimed to review and synthesize the qualitative studies on HIV-related stigma as experienced by WLWH in the U.S. to inform on the current state of stigma for WLWH and to consider the development of an intervention to help WLWH in the management of their stigma.

## Methods

All qualitative studies of women of any race, ethnicity, nationality, or class living with HIV infection in the United States were eligible for inclusion which was identical criteria to the first metasynthesis. We focused on women living in the U.S. for two reasons: there is diversity in health care systems, values, cultural norms, and attitudes toward people living with HIV around the world, and we wanted the results of this metasynthesis to be comparable to the first, to determine what progress, if any, has been made in the intervening years.

### Search strategy

A systematic and iterative search was initiated in March 2021 and repeated in May 2023 to identify all potential studies for inclusion. We began our study with the following inclusion criteria: all reports of qualitative studies published since 2004 on women in the United States living with HIV; 2004 was the year in which the last metasynthesis of qualitative findings on stigma in women living with HIV was published [7]. We conducted searches using four computerized databases (CINAHL, ProQuest, PsycINFO, and PubMed), with specific search combinations and terms refined according to the parameters established by each database. All search terms were limited to titles and abstracts in each database. We completed an initial search of PubMed with the following search terms: “HIV,“ “stigma,“ “women,“ “qualitative,“ and “U.S.“ In addition, alternative search terms referring to identified main search terms were expanded and refined to achieve comprehensive results. Alternative terminology included: *human immunodeficiency virus, AIDS, acquired immunodeficiency syndrome, seropositive, HIV-related stigma, social stigma, resilience, grounded theory, interview, focus groups, phenomenological, ethnographic, narrative, exploratory, descriptive, females, United States, USA*. We used keywords, filters, and MeSH terms whenever available to optimize search results, and university librarians facilitated the identification of additional terminology and cross-referencing of searches. Additional techniques, including manually screening reference lists, citation searching, author searches, and “berry-picking” strategies [9] were used to ensure all relevant studies were retrieved. All citations were managed using Endnote software.

### Inclusion criteria and screening

**Fig. 1:**
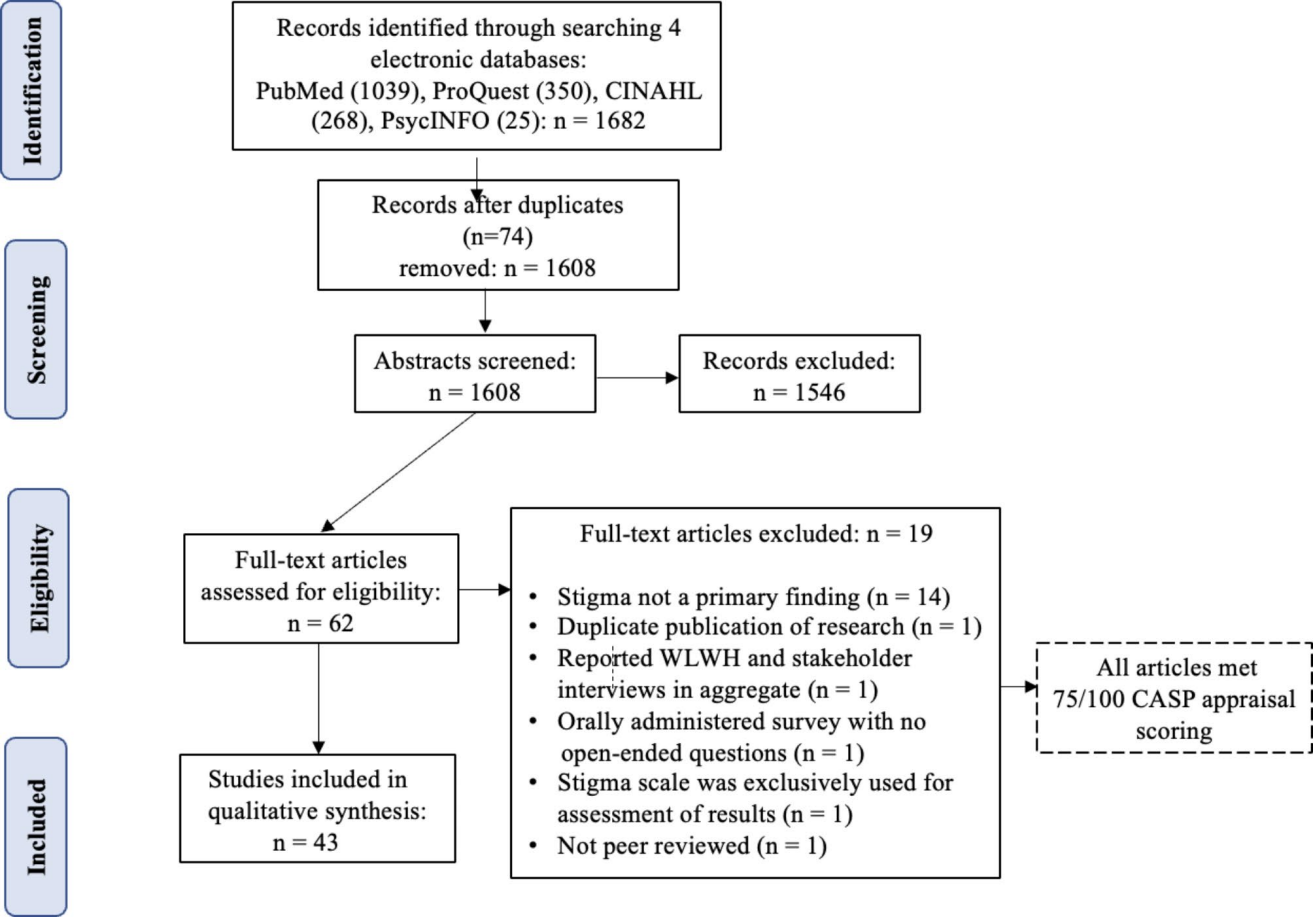
Preferred Reporting Items for Systematic Reviews and Meta-Analyses (PRISMA) diagram

We screened each article’s title and abstract to determine their relevancy before selecting them for full-text review against the inclusion and exclusion criteria. We examined all qualitative studies meeting inclusion criteria with extractable findings on perceived or experienced HIV-related stigma in which stigma was identified as a theme or distinctly separated from other elements of the research findings. We included all qualitative methodologies, as well as the qualitative findings in mixed methods studies. Additional inclusion criteria required articles to be published in English and conducted in the United States. We chose to focus on women living in the United States to account for significant variations in cultural norms, values, and healthcare delivery internationally. We specifically excluded qualitative studies with questions that were administered as open-ended prompts at the end of a structured questionnaire. Both authors independently reviewed articles identified for potential exclusion until a consensus was reached. The initial search in the four databases resulted in 1682 records. After the removal of 74 duplicates, 1608 abstracts were screened for inclusion. A total of 62 full-text articles were downloaded and assessed for eligibility, with 19 articles excluded as they did not meet the inclusion criteria. The final sample of 43 peer-reviewed qualitative studies were selected for analysis (Fig. 1).

### Critical appraisal

A team of three independent reviewers, comprised of the authors and a research assistant, critically appraised the 43 studies that met inclusion criteria for rigor and quality. Appraisals were reviewed among the three until consensus was reached. Each study was assessed to consider if the methods were appropriate and whether the findings were well-presented and meaningful. The Critical Appraisal Skills Programme (CASP) Qualitative Checklist [10] was selected as the critical appraisal instrument since the tool was devised for use with health-related research and comprehensively fulfilled the appraisal requirements for this metasynthesis. The tool consists of ten questions to assess if the aims, methodology, design, sampling strategy, data collection, reflexivity of the researchers, ethical considerations, rigor of data analysis, statement of findings, and value of the research were appropriate and clear. Each question was rated as “Yes,” “Unclear,” and “No,” with “Yes” being a score of 1, “Unclear” as 0.5, and “No” as 0. Of the 43 appraised studies, 22 received a rating of 10, 9 were rated 9.5, 11 received a rating of 9, and one study was rated an 8. However, nearly half of the studies (47.7%) did not give meaningful consideration to the relationship between the researchers and participants; data were either insufficient to make such a determination or not mentioned. None of the studies were excluded for reasons of methodological quality. Results of the critical appraisal can be found in the Appendix as Table A1.

### Data extraction

This metasynthesis of qualitative research on stigma for WLWH was extracted, aggregated, interpreted, and synthesized following the procedures outlined by [9]. First, each included study was read through in its entirety. Then, we created a descriptive table to display the summations of the articles. The following data were extracted: author, date of publication, study aims, location, published journal, methodology, data collection and interview content, sample size and characteristics, and significant themes and findings. The basic characteristics of included studies are presented in Table 1. More expansive descriptive summaries of the included studies can be found in the Appendix as Table A2.

**Table 1:** Characteristics of studies and participants

Author/Year	Aim	Location	Methodology	*N*	Mean age
Buchberg et al. (2015)	Identify factors associated with postpartum retention in care among WLWH	Houston, Texas	Mixed methods	22	28.2
Buseh et al. (2006)	Explore African American women’s narratives of LWH to understand how they experienced and responded to HIV stigma	Urban and rural Wisconsin	Narrative	29	40
Caiola et al. (2017)	Explore the social determinants of health for African American LWH by examining how mothers describe their social location at the intersection of gender, race, and class inequality; HIV-related stigma; and motherhood	Semi-urban area of North Carolina	Qualitative descriptive	18	41.5
Carr, R. & Gramling, L. (2004)	Determine the beliefs and behaviors European American women use to maintain, improve, or enhance their health after being diagnosed with HIV/AIDS	Location not disclosed	Ethnography	9	37
Cuca, Y. & Rose, C. (2016)	Examine reproductive decision-making among WLWH	San Francisco, California	Grounded theory	20	46
Dale et al. (2018)	Sought the insights of BWLWH on how they cope with multiple adversities and their thoughts on a proposed adapted intervention	Boston, Massachusetts	Not specified	30	46.5
Dale, S. & Safren, S. (2018)^1^	Shed light on the ways social support may be a resilience resource for BWLWH	--	--	--	--
Davis et al. (2021)	Examine personal HIV-stigma experiences in Latina/Hispanic and African American women using PhotoVoice	Los Angeles, California	Mixed methods	15	49.6
Davtyan et al. (2016)	(1) Identify the factors associated with WLHIV staying in or leaving IPV relationships (or ending); (2) Understand the specific roles that HIV stigma and attachment play in WLWH’s IPV relationships; (3) Learn how medical and social service providers can support WLWH to safely end IPV	San Francisco, California	Phenomenology	10	37.5
Fair, C. & Brackett, B. (2008)	Understand how HIV-positive mothers and their children experience and interpret stigma and discrimination	North Carolina	Mixed methods	8	41.5
Fernandez et al. (2022)	Understand the role of housing instability, particularly sociocultural- and stigma-related factors, and competing demands that impact daily ART adherence and care engagement	South Florida	Phenomenology	16	48.5
Fletcher et al. (2016)	Explore perspectives about reproduction and motherhood and how they were impacted by healthcare provider advice	South Carolina	Phenomenology	42	37.7
Fletcher et al. (2020)	(1) Examine resilience strategies used to cope with stressors and challenges; (2) Assess the associations of resilience with HIV health outcomes in the context of differing levels of internalized HIV-related stigma and depressive symptoms	Birmingham, AL; Jackson, MS; Atlanta, GA; San Francisco, CA	Mixed methods	76	48
Grodensky et al. (2015)	(1) Investigate the important psychosocial factors impacting older women’s living and coping with HIV infection, particularly in social and spiritual relationships; (2) Explore relationships between those factors	Southeastern U.S.	Not specified	15	57
Hampton, C. & Gillum, T. (2020)	Understand the experiences of African American WLWH/AIDS in relation to HIV-related stigma and the ways in which these experiences have affected their self-perceptions	Northeast region of the U.S.	Phenomenology	16	46
James-Borga, J. & Frederickson, (2018)	To gain a deeper understanding of the experience of LWH for older African American women	Location not disclosed	Phenomenology	10	58.9
Kempf et al. (2010)	Explore the barriers and facilitators to clinic visit adherence among HIV-positive women residing in the southeastern United States	Rural Southeast Alabama	Qualitative descriptive	39	46.1
Kim et al. (2021)	Disambiguate the additive effects of mother-child relationship quality, maternal anxiety, and maternal HIV stigma on child psychosocial adjustment with WLWH and their serostatus negative children	Georgia and California	Mixed methods	14	39
Koch et al. (2022)	Explore coping and resilience among Black women living with HIV in the Southern United States	North Carolina	Mixed methods	20	51.7
Lekas et al. (2006)	(1) To analyze the experiences of felt and enacted stigma among WLWH/AIDS and examine the extent to which they have changed over time; (2) Analyze the role of race and/or ethnicity in these women’s experiences of stigmatization	New York, NY	The qualitative portion of a community engaged study	158	36.5
Marg et al. (2020)	Understand the challenges, coping strategies, and experiences of older WLWH	Coachella Valley, California	The qualitative portion of a community engaged study	9	57
McDoom et al. (2015)	Understand how older BWLWH perceived their experiences with stigma and social support and how it either facilitated or inhibited engagement in HIV care	Boston, Massachusetts	Grounded theory	20	56.6
McMillian-Bohler et al. (2023)^1^	Explore stigma and disclosure among women living and aging with HIV in North Carolina	North Carolina	Mixed methods	2	52.2
Ojukwu et al. (2022)	Explore the facilitators and barriers of HIV treatment engagement among Black older women living in the Southeastern United States	South Florida	Qualitative descriptive	17	57.4
Peltzer et al. (2016)	To understand the everyday experiences of young African American HIV-positive women	Midwestern metropolitan area	Phenomenology	11	25
Peltzer et al. (2017)	Examine African American WLWH’s experiences of psychological distress and their use of coping strategies	Kansas, Missouri	Qualitative descriptive	22	48
Phillips et al. (2011)	Sought the meaning of WLH/AIDS in isolated, impoverished circumstances in the rural Southeast United States	South Carolina, Georgia, and Alabama	Phenomenology	39	39.75
Qiao et al. (2021)	Explore perceptions of functional wellness for WLH from the perspectives of WLH and HCPs	South Carolina	Grounded theory	20	48.9
Relf et al. (2015)	(1) Test the feasibility and acceptability of a stigma intervention for WLWH in Southeastern U.S.; (2) Compare outcomes across time in women receiving the intervention; (3) Understand the effects of HIV-related stigma on psychosocial well-being in WLWH in the Deep South	North Carolina	Qualitative descriptive	51	46.3
Rice et al. (2018)	Answer how WLWH perceive stigma associated with their co-existing social identities	Atlanta, GA, Birmingham, AL, Brooklyn, NY, Chapel Hill, NC, Chicago, IL, and Jackson, MS	Mixed methods	76	48
Rice et al. (2019)^1^	Examine effects of stigma in healthcare settings on engagement in HIV care, and potential psychosocial mechanisms for these effects (i.e., adherence self-efficacy, depressive symptoms, and coping by substance use)	--	--	--	--
Rice et al. (2020)	Explore stigma and discrimination (due to HIV, race/ethnicity, and other intersectional identities), concepts interrelated with quality of health care for Black and Latina WLWH	Atlanta, GA, Birmingham, AL, Brooklyn, NY, Chapel Hill, NC, Chicago, IL, and Jackson, MS	Mixed methods	92	Provided range
Robillard et al. (2017)	Document advice from HIV-positive African-American women to young African-American women, as described in their own cultural narratives	Columbia, South Carolina	Grounded theory	25	44.5
Sanders (2008)	Explore the meaning of pregnancy after diagnosis with HIV	Metropolitan New York	Phenomenology	9	36.5
Sangaramoorthy et al. (2017)	Explore HIV stigma, retention in care, and ART adherence in older BWLWH	Prince George’s County, Maryland	Grounded theory	35	52
Sangaramoorthy et al. (2017)^1^	Examine how stigma manifests among midlife and older BWLWH	--	--	--	--
Scott (2009)	No clear statement of aims. Study question: “What does AIDS mean?”	New Orleans, Louisiana	Qualitative descriptive and drawing	10	No mean age
Small et al. (2022)	Explore the experiences of BWLWH in healthcare settings as they relate to HIV treatment accessibility and medical mistrust	Los Angeles, California	Phenomenology and narrative	20	54
Subramaniam et al. (2017)	1) Gain an understanding of how WLWH dealt with and overcame health challenges; 2) Identify factors that contribute to sustaining resilience	Midwestern metropolitan area	Grounded theory	8	No mean age
Teti et al. (2015)	Uncover and understand women’s text and visual examples of their positive life transformations with HIV	Midwest and Northeast U.S. cities	Qualitative descriptive and photovoice	30	No mean age
Tufts et al. (2010)	Systematically collect data about the self-care experiences of WLWH	Southeastern metropolitan area	Qualitative descriptive	21	43.9
Watkins-Hayes et al. (2012)	Explicate the effects of HIV on four social domains: social support, labor force participation, childbearing and rearing, and intimate relationship	Chicago, Illinois	Grounded theory	30	36
Williams et al. (2022)	Explore the meaning and perceptions of HIV-related stigma among a sample of African American WLWH in Florida	Rural Florida	Phenomenology	13	51

^1^Data used for an additional publication

### Data synthesis

With the intention of gathering an integrated perspective while remaining faithful to the interpretive renderings in each study, we extracted each theme and main findings as reported by the authors. From there, we created a conceptual map by cataloging and comparing key themes and findings to identify overlapping attributes. We further synthesized findings by grouping each identified theme into one of three distinct categories that emerged from the articles: (1) Surviving the Intersectionality of Multiple Sources of Stigma, (2) Transcending Stigma, and (3) Responding to Stigma with Resilience. To reveal new understandings of women’s experiences with HIV-related stigma via metasynthesis, we iteratively examined the group findings until novel themes and subthemes emerged.

## Results

### Characteristics of included studies

#### Method of data collection

The data included in the reports originated from individual interviews, focus groups, or log entries. The majority of the data (81.8%) was collected through interviews. Five studies consisted of focus groups or combined focus groups and interviews. In two studies, participant intervention viewing logs and peer counseling logs were used to collect data. Five studies incorporated children of WLWH, community stakeholders, health care providers, or peer counselors, in addition to WLWH.

#### Stated methodology

Researchers in 11 reports described the method used as phenomenology, 8 as grounded theory, 6 as qualitative descriptive, 10 as the qualitative portion of a mixed methods study, 2 as the qualitative portion of a community-engaged study, 1 as narrative, 1 as phenomenology and narrative, 1 as ethnography, 1 as qualitative descriptive with photovoice, and 1 as qualitative descriptive with drawings. Three studies did not provide a specific methodological approach.

#### Sample characteristics

The publications represented a total sample of 1,118 participants. Articles that used the same data set for multiple publications were only counted once. Sample size ranged from 8 to 158 with an average sample size of 28.7. Participants’ ages ranged from 18 to 76. An aggregate mean age of 45.5 was calculated, excluding 4 articles that did not provide demographic information regarding age or exclusively offered a range. A mean of time since diagnosis was provided in 18 of the 43 included studies (41.9%) and averaged 13.6 years among participants. Of the women who participated, and in studies in which we could ascertain, 78.4% were classified as African American, 11.9% White, 8.3% Hispanic, and 1.4% as other (American Indian, Asian Pacific, “other,” and preferred not to say). Further description of the characteristics of each study can be found in the Appendix as Table A2.

### Themes

Three novel themes emerged: (1) Surviving the Intersectionality of Multiple Sources of Stigma; (2) Transcending Stigma; (3) Responding to Stigma with Resilience. Emergent themes explored a collective narrative of women first surviving the intersectionality of multiple sources of stigma, discovering non-linear pathways to transcend their stigma, and finally experiencing resilience through their transcendence of stigma. Sub-themes are presented in the order of frequency in the results.

### Frequency calculations

The frequency calculations of each finding reflect the relative magnitude of an abstracted finding within the included studies. For the validation of the themes and subthemes, we calculated the frequency for each finding by dividing the number of reports containing the finding by the total number of included articles. To establish structure and avoid under- or overweighting, themes containing frequencies equal to or greater than 20% were presented hierarchically in the results (Table 2).

**Table 2:** Themes by frequency

*Themes and Subthemes*	*Description*	*N (Frequency)*
**Intersectional Stigma**		
Healthcare Providers	Women reported stigmatizing experiences in healthcare settings.	18 (42)
Trauma and Poverty	Past and present struggles with substance use, poverty, interpersonal violence, and adverse childhood events impacted WLWH in their coping, daily lives, illness management.	17 (40)
Mental Health	WLWH experienced persistent anxiety from stigma resulting in feelings of shame, loneliness, and isolation.	16 (37)
Race and Gender	Race and gender compounded stigmatization and marginalization.	13 (30)
Family	Interpersonal stigma from family and friends resulted in abandonment, rejection, and betrayal.	10 (23)
**Transcending Stigma**		
Paradox of Disclosure	Disclosure was a source of anxiety and stress but facilitated social support and engagement in care.	20(47)
Social Support	Women built social support systems at individual, institutional, and community levels.	17 (40)
Paradox of Motherhood	Motherhood intensified stigmatization and simultaneously supported resilience.	13 (30)
Hardiness, Gratitude and Optimism	Intrapersonal qualities such as hardiness, gratitude and optimism served as sources of resilience.	12 (28)
Facing the Illness	After an initial period of denial and distress, WLWH perceived a positive life progression.	10 (23)
Spirituality	Spirituality and personal faith were integral forms of support for WLWH.	10 (23)
**Responding with Resilience**		
Accepting Oneself and Search for Personal Meaning	Constructing a positive self-identity through self-acceptance facilitated transcendence.	15 (35)
Advocacy	WLWH stressed the importance of advocacy and education.	11 (26)
Self-care	Self-care practices were integral to resilience.	10 (23)
Opposing Stigma and Transcending Constructs	Women counteracted stigma by resisting stigmatizing experiences and people and transcended negative constructs through reframing.	10 (23)
Better Able to Cope with Age	Women were better able to cope with their illness as they aged.	9 (20)

### Surviving the intersectionality of multiple sources of stigma

Intersectionality moves beyond single categories of analysis (e.g., sex, gender, race, class) to consider simultaneous interactions between different aspects of social identity and systems of oppression [11], and offers a constructive lens through which to contextualize the stigma faced by women living with HIV. Several articles in this metasynthesis incorporated intersectionality into their theoretical or guiding framework [12–15], and others introduced and discussed intersectionality [16–19]. Remaining true to the tenets of synthesizing qualitative research, themes in this metasynthesis were cataloged in the context presented by the authors and then aggregated to explore novel themes. However, we noted throughout the iterative process the interrelated nature of these various stigmas experienced across and within studies. Therefore, from within an intersectional lens, we implore the reader to view these stigmas not solely as a categorical list but to consider the complex interplay of the themes explored and acknowledge the interrelated and compounding nature of each.

#### Healthcare providers

Despite documented improvements in HIV-related stigma and discrimination, many women still reported stigmatizing experiences in healthcare settings [6, 13, 15–18, 20–26]. Cuca and Rose [6] and Peltzer et al. [24] offered the distinction that negative experiences occurred primarily with care providers who were not HIV specialists. Carr and Gramling [20] noted that discrepancies in care could vary by providers within the same institution. Several studies reported that stigma experienced in the healthcare setting was particularly damaging given the expectation that healthcare providers were responsible for and committed to their care [6, 24, 25]. Small et al. [15] noted that some women felt hurt, disengaged in care, or continued with care despite feeling unwelcome. Although prefacing results by noting that several women in the study had positive, supportive experiences with providers, Rice et al. [25] provided a detailed evaluation of HIV-related stigma in HIV care and other healthcare settings. McMillian-Bohler et al. [27] did not focus on specific instances of provider discrimination but identified several areas of additional provider engagement to help mitigate anticipated and internalized stigma.

Several studies revealed that experiences of stigma and discrimination were intensified in the healthcare setting by intersecting gender identities in pregnancy and motherhood with comments by providers reflecting judgmental reactions to their decision to have children [6, 28–30]. In addition, prevailing stereotypes and assumptions regarding behavior also raised questions from providers about their fitness as mothers [31]. Although several of the studies that examined provider-enacted stigma exclusively reflected on the experiences of Black or African American WLWH [17, 23, 24, 26] or women of color [18], results regarding provider stigma centered on women’s HIV diagnosis, as opposed to race. The intersectionality bred into their study population, theoretical frameworks, and analyses in these studies implicitly acknowledge the presence and influence of race in any interaction. However, intersectionality did not frame the discussion for stigma within healthcare settings in these articles. Dale et al. [16], Small et al. [15], and Rice et al. [13] provided narratives of racial discrimination in healthcare, while Buseh and Stevens [20] acknowledged that racial bias may have been operative in and compounding their HIV-related healthcare stigma.

#### Trauma and poverty

Traumatic life experiences were present in many narratives and linked as both a causal pathway to diagnosis and a steady state that women were operating within or recovering from. Often, women were coping with unstable and traumatic situations prior to their diagnosis. Histories of substance abuse were prevalent in many of the narratives [6, 13, 23, 30, 32–37]. For some, coping in the form of substance use followed them throughout their lives as a present state of managing or an ongoing process of recovery [24, 33, 35–39]. Past and present trauma also took many forms, with women recounting experiences of childhood trauma [6, 16, 33], intimate partner violence (IPV) [6, 16, 35], and involvement in transactional sexual relationships for basic needs [6]. In the context of ongoing IPV, stigma was noted as a source of control employed by partners to keep women in an abusive relationship [40].

In several narratives, women discussed how poverty and routine financial hardships predominated their diagnosis and day-to-day existence [6, 13, 23, 38, 41]. Phillips found that concern for basic subsistence needs precluded treatment and contributed to a cycle of dependence on family members for care [38]. When discussing adherence to care, transportation was often reported as a barrier to care [28, 38, 41, 42]. Additionally, studies discussed a lack of secure housing and homelessness as a source of strain and hardship [6, 36, 38]. Out of the 16 studies examining the impact of substance use, trauma, and poverty on experiences with HIV-related stigma, half were exclusively about the experiences of African American women [16, 23, 24, 33–35, 37, 39]. While results did not isolate race in their examination of these influences, the compounding trauma of racial discrimination [16] and higher rates of poverty and IPV [35] were acknowledged in introductions, while Subramanian et al. [39] noted increased levels of trauma and poverty for older WLWH.

#### Mental health

Many women experienced psychological distress and dysfunction because of years of internalized stigma. Struggles with self-concept started immediately following diagnosis with damaging and often lasting effects on individuals’ perceptions of their identity [19, 21, 38, 45]. Several studies noted depressive symptoms due to stigma [22, 28, 38, 43]. Internalization of demeaning stereotypes resulted in feelings of shame and self-loathing [19, 20, 29, 44]. In efforts to shield themselves from HIV-related stigma and discrimination, women frequently experienced loneliness and isolation as a result of their self-exile [23, 24, 27, 35, 43, 45]. Studies discussed the anxiety experienced over the fear of transmission to partners [33] and fetuses [30] as well as avoidance of dating and intimacy [22, 45]. Six of these studies exclusively explored the experiences of African American women [19, 20, 24, 33, 35, 44] and noted the impact of intersecting stigmas on psychological well-being [33], but reflections on negative perceptions of identity did not center on race, but rather the internalization of demeaning HIV-related stereotypes.

#### Race and gender

Discussion of race was inherent to many of the studies that exclusively reflected on the experiences of Black women (50%) [12, 14–17, 19, 20, 23, 24, 26, 33–35, 37, 39, 41, 44, 46–49]. Conversations around gender revealed prevailing stereotypes and associations with risky behaviors magnified the stigma experienced by women. However, for Black women, this stigma is further compounded by the direct association of HIV with marginalized groups. Many narratives present this marginalization and stigmatization at the intersection of gender and race [12–16, 18, 19, 48]. Gendered power dynamics and institutional racial inequities framed the experiences of women, highlighting the inextricability of gender and race in the context of HIV-related stigma. Dale and Safren [46] and Rice et al. [13] examined this societal intersection of HIV and race, finding that women were discriminated against due to their race and HIV status at multiple points at the institutional (e.g., law enforcement, health institutions) and interpersonal level from strangers and acquaintances.

Considering all studies included in this metasynthesis reflected exclusively on the experiences of women, discussion of gender and gender discrimination in the context of HIV was intrinsically present in each study. Discrimination based on gender was highlighted as a theme in several studies [12, 13, 16, 18, 19, 29, 41, 42, 48]. Studies further dissected gender-specific stigma in the context of motherhood. Although women frequently viewed motherhood as a positive force in their lives, they also felt their gendered roles as mothers amplified the stigma experienced. Prevailing associations between HIV and risky and immoral behavior stigmatized women in their reproductive roles, raising questions as to their fitness as mothers. Women reported stigmatization from providers around their reproduction choices [6] and from society more broadly that viewed them as vectors for infection [12, 29]. Given the moral stigma attached to the diagnosis, women also felt they were judged more harshly in their roles as caretakers to children, partners, and other family members [12, 16, 42].

#### Family

At the interpersonal level, women recounted the particularly damaging effects of stigma perpetuated by family and friends [6, 13, 17, 20, 21, 24, 31, 33, 38], noting the abandonment [38], rejection [33], and betrayal [24] that accompanies discrimination from individuals closest to them. Williams et al. [19] noted that despite stigma from the community being a prevalent issue, some of the most painful experiences of stigma were related to relationships with family members and friends.

### Transcending stigma

#### Paradox of disclosure

Disclosure was a frequent source of anxiety and stress for many women. Several studies discussed the psychological distress that resulted from anxiety and fear surrounding disclosure [19, 21, 23, 24, 27, 40, 41, 45, 49]. Sangaramoorthy et al. [41] described the weight of nondisclosure and secrecy as a constant burden for the women. Kim et al. [50] noted that women experienced disclosure-related stigma with most individuals, including their own children. McDoom et al. [23] and Relf et al. [45] highlighted the isolation that occurred as a by-product of reluctance to disclose. Carr and Gramling [21] and Peltzer et al. [35] discussed the paradox of trying to live a life of honesty while concealing their diagnosis and the extreme stress that resulted from this inner conflict. Similarly, Fernandez et al. [51] found this stigma challenging for women in the context of sharing living spaces with others who did not know their HIV status. Beyond the psychological effects, disclosure impeded engagement in care [26, 27, 32, 51], impacted the quality of care [25], and influenced women’s ability to promote and maintain their health [21].

Disclosure presented a quandary for many women who strategically disclosed their status to gain social support, yet such disclosure paradoxically made them vulnerable to stigma and discrimination. Still, in many narratives, selective disclosure was a source of empowerment [14, 16, 20, 23, 33]. Fletcher et al. [52] noted that choices around disclosure could profoundly influence how women conceptualized and coped with their illness. For some women, disclosure was critical in gaining social support and engaging in care [23]. For others, disclosure empowered women to share their stories to help others [33]. In some narratives, strategic nondisclosure was the source of empowerment. Buseh et al. [20] described strategic nondisclosure as “an act in defiance of HIV stigma,” during which women began to assert their rights and refuse stigmatizing reactions. Hampton and Gillum [33] told similar experiences in which women viewed nondisclosure as a source of power and protection.

#### Social support

Social support systems were integral to many women from the time of their initial diagnosis and throughout their life course, with women often drawing on multiple sources of support from communities, institutions, and individuals [6, 14, 16, 19, 20, 23, 27, 32, 38, 42, 43, 46, 47, 52]. Seeking and building trusting relationships with supportive providers was critical for women in their disease management [6, 26, 42, 46, 52]. Similarly, the benefits of peer support fostered through support groups and peer counseling were frequently discussed as a meaningful form of social support [6, 20, 39, 43, 46, 47]. Although family and friends were sometimes perpetrators of damaging and stigmatizing behavior, supportive friends [17, 32, 43, 46] and family [20, 23, 39, 46, 47, 52] were also significant sources of social support. More specifically, positive relationships with children and grandchildren [14, 16, 23, 32, 42, 46] were essential in managing their physical and mental well-being in their pathways towards transcendence. Some women also received support from religious institutions [32, 37, 52].

#### The paradox of motherhood

The paradox of motherhood and HIV are presented simultaneously in the narratives as the source of intensified stigmatization and a source of resilience. Cuca and Rose [6] and Lekas et al. [29] described the overstigmatization and intensified feelings of shame that the women felt from society and healthcare providers around their reproductive choices. Similarly, Kempf et al. [42] and Sangaramoorthy et al. [12] noted increases in anticipated and experienced stigma for women in their roles as mothers. Kim et al. [50] discussed how mothers felt intensified stigma in relation to transmitting HIV, with comments centering on judgment that they might have engaged in unprotected and promiscuous sex.

However, many studies highlighted the positive impact of motherhood. In the context of motherhood as a social determinant, Caiola et al. [14] discussed the protective effect of motherhood against other intersecting social determinants such as race, class, and gender that were negatively impacting the women’s lives. For some women, receiving an HIV diagnosis was a decisive moment in which they reevaluated their roles as mothers and took the initial steps toward recovery [28, 30]. For women who were previously struggling with their diagnosis, the pregnancy itself was a motivator to seek specialized care. Cuca and Rose [6] and Sanders et al. [30] described the self-advocacy that resulted from stigmatizing experiences with healthcare providers, after which women resisted stigma by choosing providers who were supportive of their reproductive choices and leaving those who were not. Supportive, positive relationships with children and grandchildren were also frequently noted as a motivator for women to maintain their health and remain engaged in care [14, 16, 23, 28, 32, 42, 46].

#### Hardiness, gratitude, and optimism

Several articles identified specific intrapersonal qualities such as hardiness, gratitude, and optimism as sources of resilience, finding that such traits were intrinsic to overcoming adversity associated with living with HIV and remaining hopeful for the future [12, 13, 16, 19, 33, 34, 39, 46, 52, 53]. For some women, HIV served as a reminder of their strength and perseverance and increased their feelings of resiliency and competency [12, 13, 33, 52, 53]. Williams et al. [19] found that despite the stigma experienced, women demonstrated a positive attitude that assisted in overcoming their HIV-related stigma, and most did not care to let it affect their lives. Through their struggles, women remained hopeful for the future [16, 34, 39, 45]. Some women found gratitude and hope through spirituality [39, 52], while others drew on their relationships with children and grandchildren as motivation for the future [14, 46].

#### Facing the illness

Consistent throughout the narratives, women experienced extreme emotional stress upon receiving their HIV diagnosis [19, 33, 34, 39, 41, 47, 48]. For some women, diagnosis triggered a downward spiral of denial and drug abuse [48]. For others, diagnosis resulted in a period of self-imposed isolation [34] and failure to engage in care [41]. Still, embedded in the narratives was the process during which their HIV diagnosis became the impetus for positive change [6, 30, 34, 36, 47]. Cuca and Rose [6] revealed that HIV served as a resource for basic healthcare services, case management, social workers, and support groups for women who previously lacked access. Additionally, specialized care served as a gateway to drug rehab programs and housing [6]. Similarly, Teti et al. [36] described the HIV diagnosis as a wake-up call during which women transitioned from substance abuse to recovery, crediting HIV with “saving their lives.” In motherhood, Sanders et al. [30] found that the diagnosis helped women take initial steps towards significant change and reevaluation of their roles as mothers.

#### Spirituality

Spirituality and personal faith emerged as a salient theme through which women drew strength and hope [13, 19, 20, 22, 32, 35–37, 39, 52]. Several authors described religious faith and personal spirituality as a coping mechanism [22, 36, 52]. Similarly, Buseh and Stevens [20] described women’s spirituality as a resource. Tufts et al. [37] described the practice of faith (e.g., prayer, singing in a choir, attending church) as self-care behaviors. In addition, belief in God served as a source of unconditional acceptance [32, 35] and buffered against the damaging effects of stigma [20, 22]. Personal faith and religiosity as support were most often discussed discretely from religious communities and the communal practice of religion [19, 20, 22, 35, 36, 39, 52]. As Subramaniam et al. [39] and Williams et al. [19] noted, although women sought support through prayer and their religious community, leaders and members of the community were, at times, the source of stigmatizing interactions.

### Responding to stigma with resilience

#### Accepting oneself and search for personal meaning

For many women, accepting and prioritizing the self was fundamental to building and sustaining resilience [16, 23, 34, 36, 39, 47]. Koch et al. [49] connected self-compassion as an essential element to overcoming the negative effects of HIV-related stigma and shame. Sometimes women achieved self-acceptance through disclosure. As women released themselves from the fear of disclosure, they were correspondingly released from their shame in hiding [23, 36]. Self-acceptance also facilitated continued transformation. James-Borga and Frederickson [34] found that with self-acceptance came an enhanced sense of well-being and an ongoing need to continue to grow, while Dale et al. [16] noted that as women practiced self-primacy, they were able to draw on an internalized self and sense of power.

Resilience often took shape as women searched for a sense of personal meaning in their lives. For some women, this meant constructing an identity beyond their diagnosis. By honoring the life-altering presence of HIV yet denying it further power, they were able to create a life and identity beyond HIV [36, 39]. Despite histories of trauma and stigma, women drew on an internalized sense of power and self, harnessing their struggles as proof of their resilience [13, 16, 34]. Although not yet achieved, some women remained hopeful for a positive self-concept to emerge [45] Others looked beyond themselves for personal meaning, drawing on faith [36, 37, 52], children and grandchildren [14, 46], and advocacy [19, 20, 34, 52] as sources of personal meaning.

#### Advocacy

Seeking and providing peer support was integral to many women’s experiences [16, 17, 19, 20, 34, 36, 39, 47–49, 52]. Women reinforced their sense of progress by sharing stories of their resilience while simultaneously promoting the same resiliency for others. In this sense, advocacy was a self-propelling and bi-directional force. Fletcher et al. [52] discussed the bidirectional nature of advocacy, noting that encouraging and empowering other women was integral to their resilience process. Koch et al. [49] noted the importance of service for women to support and nurture resilience in others. For Robillard et al. [47], peer advice emphasized the need for young women to value themselves and exercise their agency by protecting their sexual health. Similarly, Watkins-Hayes et al. [48] found that participants challenged narratives that construct Black women as powerless through leadership roles as peer counselors. Women also experienced the revelation of their resiliency through sharing their experiences with others during the interview process and over the course of the study [20, 36]. However, resilience through advocacy was not always experienced as an active state. After years of experiencing the deleterious effects of stigma, some women could not envision or experience a positive progression. For these women, their desires for education and advocacy focused primarily on their hope for the next generations [31].

#### Self-care

Self-care as a form of resilience was a prominent theme in many of the narratives. Considering women were sometimes facing lifelong trauma and stigma, self-care was frequently a learned behavior. After a destructive period of post-diagnosis behavior, many women came to a place of personal commitment or recommitment to their health and well-being. Adhering to medical regimens was the most noted form of self-care [16, 34, 36, 37, 41, 43, 48, 53]. Self-care also came in the form of avoiding previous unhealthy coping strategies [34, 36, 48] and pampering themselves [19, 37, 47, 48]. By acknowledging the power of self-care and their agency in performing it, women harnessed self-care as a powerful source of resilience. Engaging in self-care through activities such as pampering and adhering to treatment [37] and seeking holistic care through functional wellness [53] contributed to feelings of transcendence and resilience.

#### Opposing stigma and transcending constructs

Counteracting and opposing stigma was a challenging and ongoing process. There is an act of confrontation inherent to counteracting in which women must make two choices: first to engage, then to oppose. The amount of resilience required to achieve this act of defiance in the face of years of compounding stigma seemed daunting. Yet, throughout the narratives, women continued to make this challenging choice. Buseh et al. [20] spoke about this process during which women built the stamina to deal firmly and without evasion in an uncomfortable social situation. In healthcare settings, women expressed direct dissent with providers and left unsupportive ones [6, 25]. In some cases, the perpetrators of stigmatizing behavior were those closest to them. As women sought to create more positive social worlds for themselves, women resisted stigma by distancing or terminating relationships with unsupportive family and friends [6]. From this perspective, stigmatizing interactions and experiences were opportunities to hone their resilience rather than further defeat them.

Transcending negative constructs surrounding HIV stigma was often a complicated and non-linear process, yet essential to resilience. Caiola et al. [14] identified this process as a cognitive reframing away from the narrative of oppression towards a more positive self-assessment. In this new construct, women came to view themselves independently from their status and stigma. Several studies observed this process in which women began redefining stigma as ignorance [13, 19, 20, 22, 50]. Through this lens, they directed the fears and hostility surrounding HIV away from themselves as individuals and onto a lack of education and the perpetuation of cultural myths. Although not yet fully realized, Sangaramoothry et al. [12] and Williams [19] discussed the familiar healing powers of time, noting that women were better able to cope with negative public attitudes over time. Similarly, Relf et al. analysis revealed that as women released themselves from ownership of the stigma, they were more accepting of it as a part of their existence [45].

#### Better able to cope with age

The path towards transcendence often occurred slowly over years, and sometimes decades, of living with HIV, and the trajectory was rarely linear. Watkins-Hayes et al. [48] noted that learning to live with HIV was not only about disease management but also about coming to terms with prior experiences of pain and marginalization that may have led to their exposure to HIV. Women described periods of vulnerability and diminished confidence but also noted that over time, they came to a more frank and open awareness about HIV and the stigma surrounding it [13, 20, 36]. James-Borga and Frederickson [34] spoke to this ebb and flow of emotions experienced over the life course, ranging from extreme depression and isolation to feelings of gratitude, joy, and happiness. Naturally occurring life events such as the birth of a grandchild [41], or more decisive events such as committing to recovery [35] or securing stable housing [34], shifted perspectives towards a hopeful future. For others, acceptance and growth simply came with age. Sangaramoothy et al. [41] noted that, unlike gender and race, women perceived age as a protective factor from HIV-related stigma, finding that they were better able to cope with negative public attitudes and surrounding stigma as they aged. With time, many women were also more open to reframing their illness and accepting support [19, 37, 48].

**Fig. 2:**
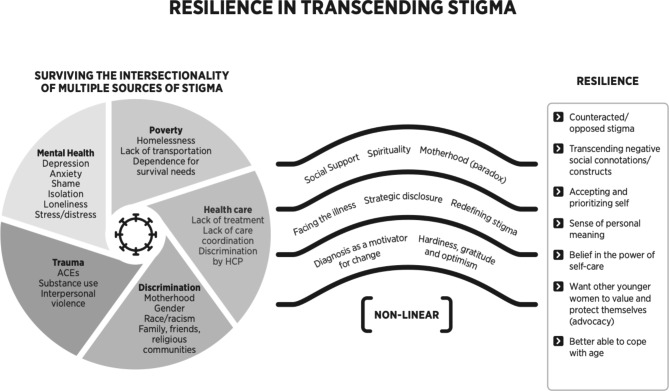
Visual representation of results

## Discussion

This metasynthesis revealed several critical changes from the first one conducted. The key findings from the first metasynthesis included the pervasiveness of both felt and enacted stigma, gender-linked intensification of HIV-related stigma, and the unending work and care of stigma management. It also included a calculus regarding the disclosure of one’s HIV status. While the second metasynthesis revealed similarities, they differ primarily in women’s abilities to find agency in dealing with stigma and to make their own determinations about the persons doing the stigmatizing.

In this current metasynthesis, women had to first survive the intersectionality of multiple sources of stigma. Although many of the same findings were present in the prior metasynthesis, they have been conceptualized differently, recognized now as interconnected social categorizations as they apply to WLWH, creating overlapping and interdependent systems of disadvantage. Many of the findings in the section on the intersectionality of sources of stigma preceded women’s diagnosis of HIV and were intensified by this diagnosis.

However, the divergence comes in women’s abilities today to transcend the stigma associated with HIV. In the first metasynthesis, the primary coping strategy was to think very carefully about disclosure; we called this the calculus of disclosure. In the current metasynthesis, while many of the emotions and effects of disclosure were the same as the first one, strategic nondisclosure was a source of empowerment and “an act in defiance of HIV stigma” [20]. Women felt a sense of agency and power in their choices regarding disclosure.

The category of responding to stigma with resilience is entirely new and was not found in the first metasynthesis. Accepting and prioritizing themselves was critical to building resilience and lead to an ongoing need to grow. Women found that they could acknowledge the presence of HIV in their lives yet deny it further power, and they could reframe their struggles as further proof of their strength. Becoming an advocate for other women reinforced the WLWH’s sense of resilience and helped women see themselves as having agency in their lives. Women also realized the importance of self-care and, in particular, the need to adhere to their medication regimens. Opposing stigma meant that women had to first engage those enacting the stigma, then choose to oppose it. Through this process, they confronted those who were stigmatizing them, such as healthcare providers and family members, and denied them further power. A cognitive reframing of their stigma helped women move to a more positive assessment of self and to redefine stigma as ignorance. In doing so, they distanced themselves from stigma and viewed themselves as separate from their HIV diagnosis. Finally, our metasynthesis revealed that as women aged, they were better able to cope with stigma.

### Limitations

There are limitations to this metasynthesis. We limited it to studies reported in English involving women living with HIV in the United States. Our primary rationale for this is two-fold. We desired to compare results to the prior metasynthesis on stigma in WLWH, which used these same parameters, and we were also cognizant of the highly varied healthcare systems and cultural responses to HIV and stigma around the world. We did not want those differences to impede our primary aim, which was to determine how women living with HIV in the U.S. were experiencing stigma.

### Implication for research

We found, in conducting this most recent metasynthesis, that the landscape for women living with HIV in the U.S. has indeed changed, and we must develop interventions that address the current state of this phenomenon. One of the most important distinctions between the two metasyntheses is that women fully demonstrated how they are successfully managing their stigma. WLWH in this metasynthesis have found a path forward. While there have been several successful interventions to help women with stigma, few stigma reduction interventions have incorporated the perspectives of the people living with HIV into their design and implementation [54]. These synthesized qualitative reflections are the collective perspectives and experiences of women surviving and thriving with HIV and can be informative to newly diagnosed women or those who have not yet found ways to deal with stigma. Given that this metasynthesis highlighted the compounding and intersecting nature of multiple forms of stigma, there is specifically a need for interventions that have strong ecological validity tailored to the intersectional nature of stigma experienced by WLWH.

### Electronic supplementary material

Below is the link to the electronic supplementary material.

Supplementary Material 1

Supplementary Material 2

## Data Availability

The data supporting the findings of this study are available from Julie Barroso. All of the articles in the metasynthesis are openly available.
