# Supplementary material for: A qualitative metasynthesis of stigma in women living with HIV in the United States
Source: Int J Equity Health. 2023 Aug 21;22:158. doi: 10.1186/s12939-023-01969-5 (PMC10441719; doi:10.1186/s12939-023-01969-5)
Supplement: Supplementary file 1 — Supplementary Material 1 [file 12939_2023_1969_MOESM1_ESM.docx]

| **Table A1.** Full descriptive characteristics of included studies | | | | | | | | |
| --- | --- | --- | --- | --- | --- | --- | --- | --- |
| Author /  Date | Title | Research Question/ Aim(s) of Study | Location/ Context | Published Journal | Methodology/Data Analysis | Data Collection/Interview Content | Sample size and Characteristics | Themes/Findings |
| 1. Buchberg, M. et al. (2015) | A Mixed-methods Approach to Understanding Barriers to Postpartum Retention in Care Among Low-income, HIV-Infected Women | “To identify factors associated with postpartum  retention in care among HIV-infected women.” | Houston, TX  Two county clinics that provide obstetric care for uninsured and underinsured HIV-infected women | AIDS Patient Care and STDs | Mixed methods  Baseline survey during pregnancy; consisting of various self-report demographics, behavioral, and psychosocial measures  Semi-structured qualitative interview at postpartum OB appointment  Analyzed using NVivo 10; developed codebook; defined codes based on themes; compared to data-derived codes | 30-minute interviews  Intended to explore women’s social and clinical experiences related to living with HIV/AIDS, in addition to the barriers and facilitators linked to their retention in care; completed the same self-report questionnaire  Conducted up to 180 days postpartum  Participants compensated $20 at baseline | (n=32) pregnant WLWH for survey; (n=22,) semi-structured interviews  18+; able to read and write in English or Spanish; in 2^nd^ or 3^rd^ trimester; intending to continue care with the county postpartum | Barriers: competing responsibilities for time, lack of social support outside of their immediate family, limited transportation access, and experiences of institutionalized stigma  Facilitators: knowledge about the benefits of adherence to care, and strong relationships with healthcare providers.  Feelings of institutionalized stigma, and high levels of depressive symptoms were identified in quantitative and qualitative results as being associated with poor retention in care. |
| 1. Buseh, A. et al. (2006) | Constrained but Not Determined by Stigma: Resistance by African American Women Living with HIV | “To explore African American women’s narratives of living with HIV to understand how they experienced  and responded to HIV stigma.” | Urban and rural Wisconsin  Interviews conducted over two years | Women & Health | Multi-staged qualitative narrative analysis  Analyzed using NVivo; analysis of AA subsample; feminist interpretive research; stigma story operationalized; followed by within-case and across-case analysis; validated by participants | 2-3 hour interviews  10 in-depth, open-ended interviews; participants narrated life since HIV diagnosis course of two years.  Broad, open-ended questions were posed in relation to specific aims.  In subsequent interviews, more specific and sensitive topics were explored in more detail (e.g., daily routines, relationships with children, work, social networks, community bonds).  Participants were compensated with $30 | (n=55) WLWH racial diverse sampling; subsample (n=29) BWLWH | Constrained by stigma: African American women described encountering HIV stigma within themselves, as acted out by others, and in the treatment they received from institutions.  Resisting stigma: enlisted support from family and spiritual communities; disclosed only at strategic times; redefined stigma as ignorance; became advocates for other BWLWH  Participants did not “become accustomed to or managed stigma; rather, they counteracted and opposed it.” |
| 1. Caiola, C. et al. (2017) | Capturing the Social Location of African American Mothers Living With HIV: An Inquiry Into How Social Determinants of Health Are Framed | “To explore the social determinants of health for African American mothers living with HIV by examining how mothers describe their social location at the intersection of gender, race, and class inequality; HIV-related stigma; and motherhood.” | Semi-urban area of North Carolina | Nursing Research | Qualitative descriptive design  Content analysis used to code interview data; frame analysis (Koenig, 2006) used to describe social location; interpretive methods and frame analysis used to examine the meaning of these constructs for participants  Photo elicitation technique used in 2^nd^ interview | In-depth interviews conducted at three time points over eight weeks.  Interview guides designed to explore the intersection of gender, race, and class inequality; HIV-related stigma; and motherhood experiences of AA mothers living with HIV; and any other SDOH identified by participants | (n=18) BWLWH  18+; self-reported HIV infection; designated race as Black/African American; able to communicate in English; had one or more dependent child(ren) between the ages of 0–18 years residing in the home; and reported eligibility for public assistance, Medicaid, or living below FPL | Content analysis: SDOH, specifically racial and gender inequity, compound HIV-related stigma  Frame analyses: (a) an emancipatory frame, marked by attempts to transcend the negative social connotations associated with HIV and socially constructed identities of race, gender, and class; (b) a maternal frame, marked by a desire to maintain a positive maternal identity and maternal-child relations; and (c) an internalized frame, marked by an emphasis on the deleterious and stigmatizing effects of HIV  The social location and health experiences of AA mothers living with HIV operates at the intersection of gender, race, and class inequality. |
| 1. Carr, R. & Gramling, L. (2004) | Stigma: A Health Barrier for Women with HIV/AIDS | “To determine the beliefs and behaviors European American women use to maintain, improve, or enhance their health after being diagnosed with HIV/AIDS.” | Location not disclosed  1995-1997 | Journal of the Association of Nurses in AIDS Care | Ethnographic methodology with repeated interviews  and observation  Interview  Data analysis was concurrent with data collection and participants were consulted to discuss, expand, and confirm findings. | In-depth interviews  Focused on participants’ health-promoting lifestyles and observation-participation  One participant was interviewed once, 3 participants were interviewed twice, and 5 were interviewed three times for a total of 22 interviews.  Audiotaped interviews took place in a variety of places chosen by the participants. | (n=9) WLWH  Purposive sampling of European American  women with HIV/AIDS | (1) stigma leads to redefinition: participants never perceived themselves in the same way  (2) the many faces of stigma: physicians advised to tell no one to mitigate being shunned and rejected (3) revealing to family and friends: family and friends were often not supportive (4) employers and co-workers: instructed not to reveal; others encouraged resignation (5) stigma and healthcare providers: many providers reacted with disgust, disdain, and fear, with physicians often being the most vocal  Experiences share rejection by family members, friends, health care providers, employers, and church members. Rejection caused by stigma affects access to health care, medication adherence, social interaction, and social support. |
| 1. Cuca, Y. & Rose, C. (2016) | Social Stigma and Childbearing for Women Living with HIV/AIDS | “To examine reproductive  decision making among women living with HIV.” | San Francisco Bay Area  2009-2010  and 2012-2013 | Qualitative Health Research | Grounded theory  In-depth interviews and participant observation  Situational analysis (Clarke, 2005) was also used as a supplement to grounded theory.  Open coding technique | 45-60 minute interviews  Later interviews took into consideration issues and trends gleaned from coding earlier interviews. | (n=20) WLWH  18+; diagnosis at least 1 year prior to participation; biologically female; English speaking  Needed to have been pregnant at least once since their HIV diagnosis, including if they were diagnosed with HIV while pregnant.  Currently pregnant women were excluded | Themes: (1) chaos, instability, and trauma: in addition to coping with HIV and pregnancy, trauma was an overarching and unanticipated theme. (2) Overt stigmatization: despite their HIV, many of the women wanted children, but experienced stigmatization related both to their HIV and to their decisions to have children. This stigmatization came from multiple sources, including healthcare providers. (3) resistance: participants demonstrated resistance to stigmatization, through building supportive communities and developing trusting relationships with HIV providers. |
| 1. Dale, S. et al. (2018) | Still I Rise: The Need for Self-validation and Self-care in the Midst of Adversities Faced by Black Women with HIV | “We qualitatively sought the insights of BWLWH on how they cope with multiple adversities and their thoughts on a proposed adapted intervention.” | Boston, MA  June 2015 - December 2016 | Cultural Diversity and Ethnic Minority Psychology | Semi-structured interviews  Coded using thematic content analysis and grounded theory; coding manual for themes developed; themes were defined and accompanied by exemplary quotes in the manual. Manual was used to code narratives in NVivo | 60-90 minutes interviews  Interviews examined (1) experiences with trauma, racism, HIV stigma, gender roles expectations (2) strategies to cope with adversities and promote medication adherence, perceptions about the need for and acceptability of a potential integrated treatment for trauma symptoms, racial discrimination, HIV discrimination,  gender related stressors, and medication adherence  Participants were compensated with $25 | (n=30) BWLWH  18+; identified as Black and/or African American; biologically female; English speaking; prescribed ART for at least the last 2 months; history of abuse/trauma (i.e., responding “yes” to “During your lifetime have your experienced trauma or abuse?”) | Participants shared (1) their experiences with trauma/abuse, racism, HIV-stigma, gender-related stress, and medication adherence.  (2) discrimination: institutional (e.g., law enforcement, health institutions), interpersonal (e.g., strangers, acquaintances). (3) gender-related stressors: (a) put other first (b) complete domestic chores (c) be lady-like  (4) coping with multiple adversities: strategies they use (e.g., social support, awareness [acknowledging systemic racism], assertiveness, selective disclosure of HIV status, and prioritizing the self). (5) connection between adversity and health: each adversity related to their medication adherence and self-care. They found ways to self-validate and practice self-primacy. Viewed children/grandchildren as motivation (6) enthusiasm for the proposed intervention. |
| 1. Dale, S. & Safren, S. (2018) | Resilience Takes a Village: Black Women Utilize Support from Their Community to Foster Resilience Against Multiple Adversities. | “To shed greater light on the ways in which social support may be a resilient resource in the lives of BWLWH. It is especially important to understand Black women’s resilience in the context of common adversities they face such as trauma/abuse, racial discrimination, HIV-stigma, and gender roles related stressors.” | Boston, MA  June 2015 -December 2016 | AIDS Care:  Psychological and Socio-medical Aspects of AIDS/HIV | Semi-structured interviews with BWLW and community stakeholders  Coded using thematic content analysis and grounded theory; coding manual for themes developed; themes were defined and accompanied by exemplary quotes in the manual. Manual was used to code narratives in NVivo | 60-90 minutes interviews  Interviews examined (1) experiences with trauma, racism, HIV stigma, gender roles expectations (2) strategies to cope with adversities and promote medication adherence.  Stakeholder interviews (1) type of organization the stakeholder worked for, and programs and services offered by the organization for PLWH and BWLWH, (2) healthy and unhealthy ways the stakeholder has seen BWLWH cope with trauma/abuse, racial discrimination, HIV stigma/discrimination, and gender-related stress. | (n=30) BWLWH  18+; identified as Black and/or African American; biologically female; English speaking; prescribed ART for at least the last 2 months; history of abuse/trauma (i.e., responding “yes” to “During your lifetime have your experienced trauma or abuse?”)  (n=15) community stakeholders | Resilience was fostered by members of their “village”. BWLWH used social support from their (1) children/grandchildren: children were instrumental in encouraging and reminding them to take their HIV medication and in making other health decisions when needed. (2) family: mothers/fathers/sibling (3) partners (4) friends/peers: both informally and in the context of support groups (5) providers: specifically in addressing mental and physical health issues beyond HIV |
| 1. Davis, K. et al. (2021) | Women of Color Reflect on HIV-Related Stigma through PhotoVoice | “To address the gaps in existing scholarship, we examined personal HIV-stigma experiences in Latina/Hispanic and African American women using PhotoVoice.” | Los Angeles-based HIV specialty center during routine clinic visits | The Journal of the Association of Nurses in AIDS Care | Interpretive phenomenological analysis  Participant narratives and photographs recorded using PhotoVoice; followed by focus groups and semi-structured interviews; a certified Spanish interpreter translated one interview  Coded using ATLAS.ti and by hand then matched to respective photographs; codes grouped into main concepts and themes | Participants were provided with a digital camera to take photographs based on experiences with HIV-related stigma.  Participants were asked to select 5-6 photographs, record the meaning in a journal, and assign a caption in preparation for a focus group or a semi-structured interview.  Then reflect on their photos during interview, provide perspectives on the definition of  stigma, causes of stigma, and the impact or consequences of stigma, as well as recommend ways to reduce stigma.  Received 3 $50 gift cards | (n=10) WLWH  18+; self-identified as AA or Latina/Hispanic; fluent in English or Spanish; experience with stigma; willingness to provide written consent | Two primary themes: (a) ignorance/lack of education and (b) cultural myths. Two sub-themes were identified in the context of stigma experienced in health care settings: (a) treatment refusal/irrational fear and (b) indiscretion with status.  The consequences of stigma included (a) depression, (b) fear of dating and intimacy, and (c) unwillingness to disclose HIV status. To cope with stigma, participants highlighted social support from family members and reliance on faith. |
| 1. Davtyan, M. et al. (2016) | Ending intimate partner violence among women living with HIV: How attachment and HIV stigma inform understanding and intervention | “1) identify the factors associated with WLHIV staying in or leaving IPV relationships (or otherwise ending the violence in such relationships); 2) understand the specific roles that HIV stigma and attachment play in WLHIV’s IPV relationships and ending IPV in their lives; and 3) learn how medical and social service providers can support WLHIV to safely end IPV in their lives.” | San Francisco, CA; two women’s HIV primary care clinics | Social Work in Health Care | Mixed methods  Baseline survey consisting of demographics; Childhood Trauma Questionnaire – Short Form; The Revised Conflict Tactics Scale Short Form (CTS2S); HIV Stigma Scale; Experiences in Close Relationships; CAGE-AID  Iterative content analysis approach (Patton, 2002); Atlas.ti 7.0 software was utilized as an annotation and note-keeping aid; 47 different codes were organized into 10 families; major themes were developed within the families and text was organized under the themes. | 60-90 minute interviews  Conducted to gain in-depth understanding of how IPV relationships ended and the impact of HIV and HIV stigma on those relationships.  Conducted up to 4 months after surveys  Open-ended and directed questions covered IPV, experiences of living with HIV and disclosure of HIV status, childhood abuse, and resource questions about who and what was helpful to participants in coping with IPV and HIV stigma.  Participants were compensated with $15 grocery card for completing the surveys and $25 grocery card after completing the interview. | (n=108) 96 cisgender and 12 transgender WLWH for survey  (n=15) interviews with WLWH who experienced IPV since diagnosed with HIV  18+; cis-and trans- gender WLHIV who reported ever experiencing IPV in adulthood and who obtained health care at one of two women’s HIV primary care clinics in San Francisco | Despite the survey data showing a mid-level range of stigma, almost all qualitative respondents stated that HIV stigma played a significant role in keeping them in an IPV relationship.  Partners used their HIV status and HIV stigma to control them and keep them in the relationship.  Substance use and attachment insecurity were prevalent among most participants.  Most survey participants utilized HIV medical and social services to a high degree and rated them as being helpful in ending IPV. |
| 1. Fair, C. & Brackett, B. (2008) | “I Don't Want to Sit by You”: A Preliminary Study of Experiences and Consequences of Stigma and Discrimination from HIV-Positive Mothers and Their Children | “To understand how HIV-positive mothers and their children experience and interpret stigma and discrimination using quantitative and qualitative methods.” | Various HIV service organizations in North Carolina | Journal of HIV/AIDS Prevention in Children & Youth | Mixed methods  Survey  Interview, open-ended questions  Transcribed interviews were coded and analyzed using Atlas.ti; two independent readers coded responses to all open-ended questions. | 1-hour interviews  The mothers’ interview included assessments of HIV-related stigma, discrimination, anxiety and depression; were asked to elaborate when they responded positively to the experiences of discrimination.  Surveys:   - HIV Stigma   Scale (Berger et al., 2001).   - Anxiety and Depression Brief Symptom Inventory - Child Behavior   Checklist   - 14 discriminatory acts   Children completed modified Stigma Scale and reading  section of the Wide Range Achievement Test-3  Mothers and children were interviewed in their homes. | (n=8) WLWH; 9 AA; 1 white *2 HIV+ mothers of child participants died before interviews were conducted  (n=10) children; ages  8-18  (n=1) uninfected  grandmother | Mothers and children perceive HIV-related stigma differently.  Children did not report any HIV-related discrimination because they did not disclose mother’s status.  Children experienced HIV-related stigma. Primarily based upon conjecture and fear of negative reactions, rather than direct discrimination.  Children upheld negative attitudes toward HIV despite intimate relationships with HIV-positive parent.  Mothers focused on the importance of education to decrease infections and increase understanding of how HIV is not spread. |
| 1. Fernandez, S. et al. (2022) | Examining Barriers to Medication Adherence and Retention in Care among Women Living with HIV in the Face of Homelessness and Unstable Housing | “To understand subjective perspectives on the role of housing instability, giving particular attention to potential sociocultural- and stigma-related factors, and competing demands that impact daily ART adherence and engagement in care.” | South Florida  August 2020 - March 2021 | Environmental Research and Public Health | Qualitative design guided by a phenomenological approach  Semi-structured interview guide developed from social ecologic framework  Data analyzed using thematic analysis and an iterative coding process with the goal of developing themes around shared participant experiences; codebook created; descriptive codes were added throughout analysis; themes were developed from descriptive codes | ~1-hour interviews  Interviews were conducted via an audio recorded telephone call due to COVID-19 social distancing requirements.  Questions were designed to gain a deeper understanding of the relationship between housing situations and ability to adhere to ART and remain engaged in care.  Broad questions about challenges around adherence and retention and probes around interpersonal and social influences.  Participants were compensated with a $60.00 Amazon gift card. | (n=16) WLWH  18 +; self- identified as a woman, LWH; able to provide consent to participate; fluency in either English or Spanish; and have self-reported experiences of homelessness or housing instability during the previous 12 months | Difficulty storing medication, disruption in routines, inconsistent access to medication and health care disruptions, privacy issues with persons with whom they live, and competing physical and mental health concerns among women living in unstable housing situations presented as significant barriers to adherence and retention in care.  Stigma and privacy presented as themes that were pervasive to the women’s experiences. They were particularly present in the context of sharing living spaces with others who did not know their HIV status. |
| 1. Fletcher, F. et al. (2016) | "She Told Them, Oh That Bitch Got AIDS": Experiences of Multilevel HIV/AIDS-Related Stigma Among African American Women Living with HIV/AIDS in the South | “To qualitatively explore perspectives about reproduction and motherhood and how they were impacted by healthcare  provider advice.” | Five South Carolina clinics or AIDS Service Organizations (ASOs)  June 2009 - July 2010 | AIDS Patient Care and STDs | Phenomenological research design  Semi-structured interview  Two coders independently analyzed interview transcripts utilizing NVivo 10 software; Data analyzed using qualitative content analysis; data-derived codes were compared to previous developed codes and finalized through consensus; codebook developed. | 90-minute interviews  Ex.“*In what ways, if any, has being HIV positive changed your relationship with your children”*  *“Please Tell me about any advice or opinions that healthcare providers have given to you about becoming pregnant; Who have you shared your HIV diagnosis with?”*  Interviews conducted at a mutually agreed upon site | (n=42) BWLWH  Ages of 18-49; of childbearing capacity; self-identified African American; female; self- reported HIV/AIDS seropositive status; English speaking; residing in South Carolina | 1) Interpersonal-stigma: women stigmatized primarily by family members and friends.  2) Community-level stigma: occurred in the context of church and public housing settings.  3)Institutional-level stigma: manifested as exclusion, stigmatization, and discrimination within the healthcare system, pharmacy, employment, and prison settings.  HIV-positive African American women living in the South are vulnerable to experiences of multilevel HIV stigma in various settings and contexts across multiple domains of life. Stigma complicated disclosure decisions and made it difficult for women to feel supported in social, professional and medical settings that are generally regarded as safe spaces for noninfected individuals. |
| 1. Fletcher, F. et al. (2020) | Resilience and HIV Treatment Outcomes Among Women Living with HIV in the United States: A Mixed-Methods Analysis | “To (1) qualitatively examine resilience strategies used to cope with stressors and challenges (2) quantitatively assess the associations of resilience with HIV health outcomes in the context of differing levels of internalized HIV-related stigma and depressive symptoms.” | Birmingham, AL; Jackson, MS; Atlanta, GA; San Francisco, CA  June and December 2015 | AIDS Patient Care and STDs | Mixed methods  Interview  Study utilized data collected from Women’s Interagency HIV Study (WIHS)  Analyzed transcripts using NVivo 10; qualitative content analysis, using both inductive and deductive approaches; draft codebook developed before conducting interviews; data derived codes used for broader perspective; codebook used to analyze emerging themes, patterns, and perspectives. | Interviews lasted ~73 min.  Explored (1) the influence of stressors and challenges on women’s overall health and well-being and (2) the role of positive coping mechanisms and resilience strategies to combat adversity.  Conducted in private rooms by female researchers | (n=76) WLWH  18+; enrolled in the Women’s Adherence and Visit Engagement (WAVE); enrolled in the WIHS cohort study; English speaking; able to provide written informed consent | Resilience strategies used to cope with stressors and challenges, included (1) intrapersonal traits and states (i.e., hardiness, acceptance, gratitude); (2) interpersonal and institutional resources (i.e., social support from multiple sources, including family, friends, other WLWH and health care providers); and (3) active engagement with spiritual and/or religious practices.  Social ties to other individuals, groups, and the larger community can aid WLWH who often cope with multiple stressors (both HIV and non-HIV related), challenges, and adversities; active engagement with interpersonal (i.e., friends and family) and institutional networks (i.e., health care clinics, WIHS research study, and religious institutions) engendered a sense of gratitude and thankfulness for resources and social support. |
| 1. Grodensky, C. et al. (2015) | "I Should Know Better": The Roles of Relationships, Spirituality, Disclosure, Stigma, and Shame for Older Women Living with HIV Seeking Support in the South | “To (a) investigate the important psychosocial factors impacting older women’s living and coping with HIV infection, particularly in social and spiritual relationships, and (b) explore relationships between those factors.” | Public-hospital-based infectious diseases clinic in  Southeastern U.S.  May 2006 – November 2006 | Journal of the Association of Nurses in AIDS Care | Semi-structured, in-depth interviews  Generated a list of codes through open coding; grouped the list of codes into conceptual categories or themes; applied the codebook to code two randomly selected transcripts using NVivo 9; resolved discrepancy and revised as needed; final refinements to the codebook were made after applying a similar approach to five more transcripts. The remaining eight interviews were independently assigned codes. | ~1-hour interviews  Interviewer conducted continuous comparative analysis between transcribed interviews in groups of five, through which commonalities in the interviews were identified, categorized, and compared.  Guide was “designed to elicit information from the women on psychosocial aspects of their lives, particularly related to the diagnosis and management of their HIV, that would yield a thematic description of older women’s experiences living with HIV.” | (n=15) WLWH (13AA, 2 Caucasian)  50+; HIV-infected; English-speaking | Participants discussed three types of relationships that were sources of potential or actual support in their lives: (a) family and platonic relationships, specifically from daughters; (b) romantic partnerships: varied among participants (c) relationships with a church community and with God. Within each type of relationship, salient themes emerged that reflected two general issues: (a) the types of support received, and (b) factors that helped or hindered accessing that potential source of support.  Personal spirituality emerged as a consistent theme, not in the form of support from religious institutions.  Social stigmas that older HIV+ women experience impede disclosure, emotional health, and adherence to treatment. |
| 1. Hampton, C. & Gillum, T. (2020) | 'Today I Feel Strong': African American Women Overcoming HIV-related Stigma | “To provide pivotal information regarding the unique experiences of African American women living with HIV/AIDS in relation to HIV- related stigma and the ways in which these experiences have affected their self- perceptions.” | Large medical center in a mid-sized city in the Northeast region of the U.S.  Recruitment from December 2018 – April 2019 | Psychology & Health | Phenomenological approach  In-depth, semi-structured interviews  Coded in NVivo 12; research team independently coded schemes then compared; meaningful themes extracted; themes were presented to a subset of participants for authentication | Interviews ranged from 22-100 minutes  16 interviews explored the lived experience as HIV positive African American women.  Received a $20 gift card | (n=16) BWLWH  18+; self-identified as Black or African American; English speaking; biologically female; engaged in HIV/AIDS treatment at the medical center | Four themes: (1) Increased vulnerability: women recounted experiences of childhood trauma, and maladaptive coping skills (i.e., substance use) and how these experiences increased their risk of infection. (2) processing the diagnosis of HIV/AIDS: women described similar trajectories of understanding their diagnosis in relation to their preconceived notions of the illness and the internalization of what this disease meant in their lives physically and psychologically, from initial diagnosis to present day.  (3) surviving HIV/AIDS: four sub-themes: managing illness, disclosure, psychological well-being, and self-perception.  (4) quality of life: women described persistent fear of transmission in intimate relationships and among family members, coping with family rejection and HIV-related discrimination, coping with feelings of sadness and loneliness, and ways they were able to overcome adversity and have optimism |
| 1. James-Borga, J. & Frederickson, K. (2018) | The Voices of Older African American Women Living with HIV Disease | “To gain a deeper and more holistic understanding of the experience of living with HIV disease for older African American women.”  “We sought to use the voices of women to explicate their daily world experiences, free from preconceived researcher priorities or assumptions.” | Location not disclosed | Journal of the Association of Nurses in AIDS Care | Phenomenological approach  Unstructured interview  Field notes were constructed describing the environment, participant body language and/or affect, and researcher reflections; their own words were used to create individual descriptive summaries of each story; commonalities were identified grouped into categories and sub-categories; emerging themes were identified to uncover the essence of experience. | 60-90 minute interviews  The phenomenological question: *Please tell me, what is it like (for you) to be living with HIV disease?*  Interviews conducted in participants’ homes or private clinic setting.  $15, $20, and $25 gift cards given for each consecutive interview. | (n=10) BWLWH  50+; self-identified as Black and/or African American; English speaking;  currently receiving care at the health center | Seven themes: (a) knowledge as empowerment, (b) concealing while revealing, (c) hypervigilant awareness regarding HIV stigma, (d) maintaining relationality, (e) caring while being cared for, (f) emotional ebbs and flows, and (g) self-transcending and becoming. “Self-transcending and becoming” were central to each.  Maintaining connection with others allows older African American women living with HIV to rise above the complexities of their health care issues, reach out beyond themselves, and achieve well-being. Nurses have a role in facilitating this process  Women desired to obtain and share knowledge about HIV disease with their peers, health care providers, and the public. |
| 1. Kempf, M. et al. (2010) | A Qualitative Study of the Barriers and Facilitators to Retention-in-care Among HIV-positive Women in the Rural Southeastern United States: Implications for Targeted Interventions | “To explore the barriers and facilitators to clinic visit adherence among HIV-positive women residing in the southeastern United States.” | Twenty-three predominately rural counties in Southeast Alabama | AIDS Patient Care and STDs | Descriptive inquiry approach  Focus groups; broad, open-ended questions; sociodemographic survey prior to group  Qualitative content analysis; data coding and sorting was facilitated by QRS NVivo 8 software; two investigators independently coded and developed schema; reviewed with team | 2-hour focus groups  Ex. *Describe your experiencing in keeping your health care appointments.*  Four focus groups typically consisted of 6-10 but ranged in size from 5-16.  Conducted in rooms at or close to the co-operating clinics; transportation provided  $20 cash incentive | (n=40) WLWH; (37AA; 3 white)  19+; female; HIV positive and currently in care at one of the four participating HIV outpatient clinics; English speaking | Themes: (a) trustful patient/provider relationships increase the likelihood of retention in care; (b) organizational structure and location of clinics can impede access to care mediated through transportation and opportunity costs; (c) transportation costs can prevent women from attending clinic appointments if funds are not available to subsidize lack of transportation, and (d) stigma is still alive after 28 years into the HIV epidemic and impacting women seeking care. |
| 1. Kim, S.-J. et al. (2021) | Maternal HIV Stigma and Child Adjustment: Qualitative and Quantitative Perspectives | “To disambiguate the additive effects of mother-child relationship quality, maternal anxiety, and maternal HIV stigma on child psychosocial adjustment with MLH and their serostatus negative children.” | Georgia and California | Journal of Child and Family Studies | Mixed methods  Baseline survey consisting of demographics; Stigma Scale (Sayles et al., 2008), Generalized Anxiety Disorder-7 (GAD-7) questionnaire (Spitzer et al., 2006); Conflict Behavior Questionnaire short form (CBQ; Prinz et al., 1979).  Semi-structured interviews  Qualitative coding was completed by four coders using NVivo; examined transcripts for themes of ‘stigma’ (deductive) and subthemes (inductive) | ~75-minute interviews  Interviews with these mothers and their children were conducted separately.  Interviews were developed in line with the larger study goals of mothers’ disclosure of their HIV status, parenting, and disclosure. No specific queries addressed stigma.  Compensated with $60 cash. | (n=181) mothers LWH and one of their 6- to 14-year-old children.  Interviews  (n=14) mothers WLH  (n=13) children of WLH  English or Spanish speaking; mother’s confirmed HIV/AIDS diagnosis; being the primary caregiver of an HIV-negative child between the ages of 6-14; the child being unaware of mother’s HIV status (at the time of quantitative baseline data collection) and having no mental health or developmental disorders that could impact their ability to respond to interviewing. If a mother had more than one child that met the inclusion criteria, one of the children was randomly selected for participation. | A theme of stereotype stigma emerged, which included subthemes of transmission stigma and misinformation stigma.  Stereotype stigma: Mothers raised concerns about explicit negative judgment about HIV from others or their community.  Transmission stigma: Mothers discussed stigma related to negative perceptions of transmitting HIV. Most comments centered on the perception that the mother might have engaged in unprotected and promiscuous sex.  Misinformation stigma: Mothers discussed stigma related to inaccurate or lack of knowledge in others.  The responses from the mothers suggested that they experience disclosure-related stigma with most individuals, including their own children.  Maternal HIV stigma is significantly associated with child adjustment difficulties. Qualitative reports of HIV stigma are consistent with ones from over 15 years ago. |
| 1. Koch, A. et al. (2022) | Exploring Resilience Among Black Women Living With HIV in the Southern United States: Findings From a Qualitative Study | To explore coping and resilience among Black women living with HIV in the Southern United States. | North Carolina  Women’s Interagency HIV Study (WIHS) site  July - October 2016 | Journal of the Association of Nurses in AIDS Care | Secondary analysis of qualitative data from a sequential mixed methods study.  Codebook was developed a priori, using the elements of the Adaptive Leadership Framework for Chronic Illness (ALFCI).  Secondary set of codes focused on stigma and disclosure and HIV engagement in care, ART adherence, medical distrust, discriminatory experiences, and mental health challenges.  Team members worked in dyads to apply the ALFCI codes to codebook then added codes to NVivo. | To explore theoretical pathways developed in quantitative phase, three stories were developed to explore domains of interest; a set of standardized probes associated with each slide were used to guide the interviews.  Participants received $75 in compensation, $25 for transportation costs and $50 for their involvement in the study. | (n=20) BWLWH  18+; spoke English; able and willing to provide written informed consent | Six major themes highlighting coping and resilience: self-acceptance, disclosure, social support, self-compassion, will to live, and service.  Self-compassion: linked to coping with and addressing HIV-related stigma and shame and internalized HIV-related stigma. Lack of self-compassion was conversely related.  Disclosure: Secrecy was necessary to avoid stigma. Some participants attributed shame and HIV-related stigma as drivers of lack of HIV disclosure.  Service: Mothers did not want their children to feel the stigma they had felt or to experience the shame that had plagued their lives. They wanted to provide support to nurture resilience in others. |
| 1. Lekas, H-M et al. (2006) | Continuities and Discontinuities in the Experiences of Felt and Enacted Stigma Among Women With HIV/AIDS | “To analyze the experiences of felt and enacted stigma among women with HIV/AIDS and examine the extent to which they have changed over time; analyze the role of race and/or ethnicity in these women’s experiences of stigmatization.” | New York City, NY  October 1994-November 1996 (pre-ART)  March 2000 - April 2003 (post-ART) | Qualitative Health Research | The qualitative portion of a community-engaged study  In-depth interview  Thematic content analysis developed by authors; two research teams (each consisting of 2 members) coded 36 interviews; compared coding themes; scheme tested by 30 additional interviews; final scheme for both eras applied to ATLAS.ti.; time comparisons made within each racial/ethnic group | 4-hour interviews often completed in two 2-hour sessions  158 interviews from two matched samples; matched on race or ethnicity, disease stage, age, drug-use; (79 pre-ART; 79 post-ART interviews)  Participants completed a set of psychosocial measures and an interviewer-administered questionnaire for demographic and medical history; interviews completed in subsequent meetings.  Interviews explored different adaptational challenges confronting women living with  HIV/AIDS; toward the end of interview, participants were also asked to discuss how their race/ethnicity and/or their gender has influenced their lives as women with HIV/AIDS. | (n=158) WLWH; (79 pre-ART; 79 post-ART interviews)  Ages 20-45; lived in the New York City metropolitan area; if African American or White, were native born and non-Hispanic, or, if Latina, were Puerto Rican and had lived in the U.S. ≥4 years; had completed an 8^th^ grade education; had not used drugs by needle in the past 6 months  Pre-ART sample consisted of approximately equal distribution of race and disease stage. | Stereotypes as a source of stigma have remained unchanged over time; incidence of enacted stigma against women decreased in frequency and intensity over time; internalized stigma remained unchanged.  Data from the two eras revealed that the acts of stigmatization against women that were triggered by misconceptions about the ways in which HIV is transmitted persisted in the HAART era.  Both time periods and across racial/ethnic lines revealed that internalization of the demeaning stereotypes of people with HIV/AIDS caused women feelings of self-loathing, shame, and psychological distress and often leading to self-exile.  Noted improvement in provider discrimination and stigmatization in ART era. |
| 1. Marg, L. et al. (2020) | "We Are Becoming Older Women and Then We Have Two Stigmas": Voicing Women's Biopsychosocial Health Issues as They Age with HIV | “To understand the challenges, coping strategies, and experiences of older WLWH.”  Research question: “What are the major health priorities for women who are aging with HIV?” | Coachella Valley (CV) of California’s Riverside County | Journal of Women & Aging | The qualitative portion of a community-engaged study  Focus group  Analyzed using the rigorous and accelerated data reduction (RADaR) technique (Watkins, 2017).  Created an all-inclusive data table; research question developed; salient segments were coded; RADaR technique used to further condense table into themes; codes assigned to remaining data | 90-minute focus group  Community engaged approach:  questions designed by PLWH and encompassed three main areas, including major health issues (e.g., “*what are the top major health issues affecting people who are aging with HIV?”*), sources of resilience that promote healthy aging with HIV (e.g., “*what are some of the resiliencies that allow people to age healthily with HIV?”*), and priority research topics related to HIV and aging (e.g., “*what should be the top priority topics for research on HIV and aging in the Coachella Valley?”)* | (n=9) WLWH  50+; female | Four themes: (1) mental health: women experienced isolation and depression due to stigma over aging and body image (2) HIV comorbidities: concerns related to major health issues that occur alongside, and often interact, with HIV (3) resiliencies: participants emphasized the importance of social support as a major source of resilience.  (4) social determinants of health: women faced structural barriers as they age with HIV |
| 1. McDoom, M. et al. (2015) | How Older Black Women Perceive the Effects of Stigma and Social Support on Engagement in HIV Care | “To gain an in-depth understanding of how older black women perceived their experiences with stigma and social support and how it either facilitated or inhibited engagement in HIV care.” | Metropolitan  Boston area  February and September  2011 | AIDS Patient Care and STDs | Qualitative analytic techniques informed by grounded theory  In-depth,  semi-structured interviews  First three transcripts coded by team to develop codebook; support, stigma, and engagement in care were identified and a set of analytic codes that reflected these domains developed. | Interviewed lasted as long as participants were willing to talk; ranged from 26 min to 2h 45 min; median time of 51 min.  Nine main questions regarding how social support and experiences of stigma affected their ability to engage in HIV; follow-up questions probing topics that emerged during the interviews.  $25 gift card to local store | (n=20) BWLWH  50+; female; self-identified as Black or African American; able to understand and speak English; currently in HIV care in Metropolitan Boston | The interconnectedness of disclosure, stigma, and social support related to engagement in care was dominant across the women’s narratives. Often portrayed as reluctance to disclose and subsequent loss of social connections that could facilitate engagement in care. The fear of being ‘‘outed’’ as HIV-positive and negative experiences with being stigmatized inhibited their ability to find positive social support. As women began to manage the impact of potential stigma, many were able to disclose their status and obtain support that facilitated engagement in care. |
| 1. McMillian-Bohler, J. et al. (2023) | Examining Stigma and Disclosure Among Women With HIV in the Southern United States: Qualitative Study Guided by the Adaptive Leadership Framework for Chronic Illness | Through the lens of the Adaptive Leadership Framework for Chronic Illness the study aimed “to explore stigma and disclosure among women living and aging with HIV in North Carolina, a state within the geographic region of the United States South.” | North Carolina  Women’s Interagency HIV Study (WIHS) site  July - October 2016 | Journal of the Association of Nurses in AIDS Care | Mixed methods  NVivo 12.0 used to code data.  Codebook developed using the elements of the Adaptive Leadership Framework for Chronic Illness (ALFCI).  Secondary set of codes focused on stigma and disclosure and HIV engagement in care, ART adherence, medical distrust, discriminatory experiences, and mental health challenges.  Three team members immersed themselves in the data and met every other week for ~6 months. | To explore theoretical pathways developed in quantitative phase, three stories were developed to explore domains of interest; a set of standardized probes associated with each slide were used to guide the interviews.  Participants received $75 in compensation, $25 for transportation costs and $50 for their involvement in the study. | (n=22) BWLWH  18+; spoke English; able and willing to provide written informed consent | Results were categorized by technical challenges that could be easily identified and addressed, and technical work, which were actions that could be taken by providers.  Adaptive challenges that require new skills or a change in beliefs were identified. Adaptive work to be completed by WLWH was identified.  WLWH face many stigma-related technical and adaptive challenges, which can be addressed through the initiation of adaptive, technical, and collaborative work and adaptive leadership. |
| 1. Ojukwu, E. et al. (2022) | A Qualitative Study on the Social Determinants of HIV Treatment Engagement Among Black Older Women Living With HIV in the Southeastern United States | To explore the facilitators and barriers of HIV treatment engagement among Black older women living in the Southeastern United States. | South Florida  Ambulatory care center/ HIV clinic at a hospital  April - October 2017 | Journal of the Association of Nurses in AIDS Care | Descriptive qualitative approach  Two research team members inductively synthesized transcripts using a directed content analysis approach. A codebook was created from which themes and subthemes were | 45-60 minute interviews  Open-ended questions explored HIV treatment engagement and related intrinsic and extrinsic influencers. | (n=17) BOWLWH  50+; self- identified as Black; spoke and read in English or Spanish; self-reported living with HIV | Used the socioecological model (SEM) (McLeroy et al., 1988) to analyze barriers and facilitators to treatment and engagement. Themes at the intra-, interpersonal, community, and societal levels were explored. 14 themes were developed.  At the community level, HIV-related stigma and fears of disclosure inhibited engagement.  HIV treatment engagement among BOWLH is affected, both positively and negatively, by numerous social determinants of health at all levels of SEM. |
| 1. Peltzer, J. et. al. (2015) | Infected Lives: Lived Experiences of Young African American HIV-Positive Women | “To understand the everyday experiences of young African American HIV-positive women.”  Research Question: What is the lived experience of young African American HIV-positive women? | Midwestern metropolitan infectious disease clinic | Western Journal of Nursing Research | Hermeneutic phenomenological approach  Primary researcher developed narratives; one pattern with four inter-related themes emerged through iterative process. | 45-90 minute initial interviews; 30-90 minute second interviews  Initial unstructured audiotaped interview began with the prompt:  *Please tell me a story that describes your experience*  *of living with HIV infection.*  A second follow-up interview was conducted to clarify and expand on salient points.  Only initial interview data was used due to loss to follow-up.  Participants compensated $40 | (n=11) BWLWH    Ages 18-35; African American race; HIV-positive serostatus; ability to speak and read English | Four themes: (a) Living alone with HIV: women shielded themselves from the public, the community, friends, and even to ensure safety  (b) Living with unresolved conflicts: externally strong, but internally fragile; (c) Living with multiple layers of betrayal; and (d) Living in the everydayness of HIV |
| 1. Peltzer, J. et al. (2016). | A Qualitative Description of HIV-Infected  African American Women’s Experiences of  Psychological Distress and Their Coping Strategies | “To examine HIV-infected African American women’s experiences of psychological distress and their use of coping strategies.” | Kansas, MO  Internal medicine clinic in a metropolitan city in the southwest region of Kansas; infectious disease clinic in eastern city in Missouri | Journal of the Association of Nurses in AIDS Care | Qualitative descriptive  Two semi-structured interviews  Descriptive statistics used to analyze demographic data; inductive content analysis used to analyze narratives.  The narratives were entered into NVivo;  meaning units extracted; labeled into codes then categorized. | 90-minute initial interviews;  30-90 minutes follow-up interviews  Ex: *Tell me about the feelings you experienced at any time point after your diagnosis of HIV.* Additional questions included: *How would you describe these feelings? How do these feelings affect your daily life? How do you cope with these feelings?*  15 meetings in participants’ home; 3 in private ambulatory care room; 4 at local library  $25 gift card | (n=22) BWLWH; (n=10) from metropolitan city in southwest KS; (n=12) from eastern city in MO    18+; African American race; HIV-positive serostatus; ability to speak and read English; and self-reported psychological distress | Four themes: (1) psychoemotional suffering: experiences of pervasive, negative emotions and thoughts affected daily lives; (2) contextual factors (e.g., poverty, addiction) influenced the women’s distress before and after diagnosis; (3) HIV-related stigma perpetuates isolation: resulted in profound loneliness; and (4) creating a safe haven: personal strategies employed by the participants to ameliorate psychological distress    HIV-related stigma was reported as the most concerning factor that influenced participants’ experiences of suffering. |
| 1. Phillips, K. et al. (2011) | Social Context of Rural Women with HIV/AIDS | “Choosing a phenomenological stance toward the data, we sought the meaning of living with HIV/AIDS in isolated, impoverished circumstances in the rural Southeast.” | South Carolina, Georgia, and Alabama  Ten community-based HIV/AIDS service organizations serving rural areas of South Carolina, Georgia, and Alabama | Issues in Mental Health Nursing | Adapted the phenomenological methodology described by Thomas and Pollio (2002)  Qualitative data drawn from a larger, 3-year longitudinal parent study  Peer counselor notes discussed in interdisciplinary research groups; notes reflected upon separately to capture the lived experience each before exploring commonalities with other women as the analysis continued to unfold; 39 logs examined | 30-60 minute peer counseling sessions  Nine peer counseling sessions over a six-month period  Sessions were scheduled every 2-3 weeks until all nine sessions were completed.  During each session, counselors  (1) assessed the issues of concern, (2) assisted in identifying and accessing available resources and services, (3) helped to establish priorities, and (4) identified appropriate strategies for addressing concerns | (n=39) WLWH  21+, peer counselors; HIV+ for at least three years; drug- and alcohol-free for at least five years; and without physical or mental symptoms that could interfere with their ability to fulfill their responsibilities to assigned clients | Themes: (1) struggle/effort: instability is the norm; (2) stigma/hiding: the stigma associated with being HIV+ resides in both the interpersonal and social systems; (3) loss/depression: the women’s loneliness is compounded by loss of physical integrity as well as the death and desertion of loved ones; and (4) Independence/dependence: participants were engaged in cycle of dependence and independence related to basic subsistence needs.  Having HIV/AIDS was not the predominant concern of the women  in their day-to-day lives. Focus was rent, utility bill, or conflict with family members or partners. |
| 1. Qiao, S. et al. (2021) | Perceptions of Functional Wellness in Women Living with HIV in South Carolina, United States: Voices from Both Patients and Providers | “To address knowledge gaps by exploring the perceptions of functional wellness for WLH from the perspectives of both WLH and HCPs.” | South Carolina | AIDS Care | Grounded theory  Conducted using NVivo 11; two coders independently coded each transcript based on the codebook; disagreements were discussed and resolved; representative and verbatim quotes were selected to illustrate key findings. | Open-ended questions asked following an interview guide  Interviews conducted in private rooms  $25 gift card given | (n=20) WLWH  18+; living in SC; confirmed diagnosis of HIV or AIDS.  (n=10) Health care providers (HCP)  18+; providing HIV-related care and service in SC | Themes for WLWH: (1) living and functioning: highlighted as the basic meaning of wellness; (2) resilience: many women, particularly those who have had an HIV diagnosis for a relatively long time, perceived functional wellness as resilience.  Themes for WLWH and HCP: (1) prevention: both HCPs and WLH viewed comorbidity prevention, especially chronic disease prevention, as a component of functional wellness; (2) self-care; (3) social engagement: discussed as a source of life meaning and self-worth in the description of wellness. |
| 1. Relf, M. et al. (2015) | Voices of Women Facing HIV-Related Stigma in the Deep South | (1) “Test the feasibility and acceptability of a technologically delivered stigma intervention for women with HIV in the Southeastern United States.”  (2) “compare outcomes (i.e., internalized stigma, self-esteem, and coping self-efficacy) across time in women receiving the intervention.” (3) “understand the effects of HIV- related stigma on psychosocial well- being among women with HIV living in the Deep South.” | North Carolina  Six sites providing HIV-oriented medical services to women across central North Carolina, including women from urban and rural locations | Journal of Psychosocial Nursing | Qualitative descriptive  The intervention was conceptualized to work via *narrative transportation*, (Green, 2004; Green&Brock,2000; Green, Brock, & Kaufman, 2004)  Team discussed narratives; critiqued findings; identified themes and compared to the extant literature; compared experiences of women with HIV by age, race/ethnicity, and years since diagnosis. | 45-minute video  5 vignettes: (1) defined stigma (2) examined potential responses to being stigma­tized (3) assessed factors and processes to contemplate in relation to disclosing to children, family, and friends (4) discussed planning a future as a woman with HIV, including engaging in intimate relationships.  Women randomized to receive the intervention were asked to watch the video in its entirety a minimum of once per week for 4 weeks, then as they desired during weeks 5 to 12.  All participants completed questionnaires related to internalized stigma, self-esteem, and coping self-efficacy at baseline and 30 and 90 days.  Intervention arm completed viewing log. | (n=99) WLWH; (n=48) con­trol; (n=51) intervention  HIV Stigma Scale score ≥40  Moderate to high levels of internalized HIV-related stigma at the time of enrollment | Stigma of HIV/AIDS: individual and aggregate effects of the various forms of HIV-related stigma threatened the self-concept, resulting in secrecy and isolation and making it difficult or impossible to disclose one’s serostatus.  Threatened self-concept: influences his/her identity, body image, self-esteem, and role performance, which impacts decision making and relationships.  Living with a secret: women kept their HIV status a secret, anticipating stigma if others found out.  Experiencing isolation: a frequently self-imposed process to maintain self-concept.  Thinking about the future: Although women experienced, anticipated, or internalized stigma, they also contemplated a future. |
| 1. Rice, W. et al. (2018) | Perceptions of Intersectional Stigma Among Diverse Women Living With HIV in the United States | “To answer the research question: How do U.S. women living with HIV perceive stigma associated with their co-existing social identities? | Five WIHS sites located in  Atlanta, GA, Birmingham, AL, Brooklyn, NY, Chapel Hill, NC, Chicago, IL, and Jackson, MS  Part of an exploratory mixed methods  sub-study to the Women’s Interagency HIV  Study (WIHS)  June - December 2015 | Social Science & Medicine | Mixed methods  In-depth, semi-structured interviews  Transcripts coded and analyzed according to Braun & Clarke's steps of qualitative thematic analysis (2006); team of seven researchers developed a list of codes and sub-codes after data collection; four-person team of coders developed a coding framework through an iterative process and applied codes using thematic analysis methods in De-doose. | Interviews lasted 73-minute on average.  Interview guide facilitated discussions around HIV-related stigma other types of stigmas and discrimination using open-ended questions.  Ex. *“People experience stigma and discrimination based on many other things besides HIV, including race, sexual orientation, using alcohol or drugs, having mental health challenges, or engaging in sex work. Can you describe any experiences you have with discrimination based on other parts of who you are?”*  $30 cash incentive | (n=76) WLWH  18+; English Speaking | Women perceived interrelated forms of social marginalization as coming from multiple sources: communities, interpersonal interactions, and within systems and structures.  Alongside HIV-related stigma, participants most commonly discussed stigma related to their gender, race, and socioeconomic status.  Other forms of stigma that emerged included stigma related to  incarceration history, weight and less commonly stigma attributed to mental health, substance use, sexual orientation, age, disability, and sex work. Intersecting forms of stigma were often discussed by the interviewees in the context of one another, and at other times in isolation. |
| 1. Rice, W. et al. (2019) | A Mixed Methods Study of Anticipated and Experienced Stigma in Health Care Settings Among Women Living with HIV in the United States | “To examine effects of stigma in health  care settings on engagement in HIV care, and potential psychosocial  mechanisms for these effects (i.e., adherence self-efficacy, depressive symptoms, and coping by substance  use) suggested by prior literature.” | Five WIHS sites located in  Atlanta, GA, Birmingham, AL, Brooklyn, NY, Chapel Hill, NC, Chicago, IL, and Jackson, MS  Part of an exploratory mixed methods  sub-study to the Women’s Interagency HIV  Study (WIHS) | AIDS Patient Care and STDs | Mixed methods  In-depth, semi-structured interviews  A four-person team of coders developed a coding framework through an iterative process and applied codes using thematic analysis methods in De-doose. | Interview prompted discussion around barriers and facilitators related to HIV treatment adherence, including perceptions and experiences of stigma from various sources.  Ex. *‘‘In what areas of life (e.g., health care, work, family) have you experienced stigma or discrimination?’’* and *‘‘How have the negative ways that women living with HIV are viewed or treated by others impacted your life?’’* | (n=76) WLWH  18+; English Speaking | Themes: expression of (1) *assumptions* and (2) *judgment* by health care providers about the participant’s risk behavior, health-related decisions, or engagement in health care; (3) *lack of compassion* in participant–provider interactions; (4) *insufficient care*, characterized by failure to meet medical needs or inattention to medical concerns; (5) *unnecessary precautions*, including excessive control measures such as lack of touch, use of double gloves, avoidance of physical proximity, isolation measures; (6) *violation of privacy* via voluntary or involuntary disclosure of participants’ status or other lack of health information protection;  and (7) *lack of autonomy*, including  control or influence over medical decisions or disrespect for the participant’s health care desires. |
| 1. Rice, W. et al. (2020) | Quality of Care for Black and Latina women Living with HIV in the U.S.: A Qualitative study | “To explore stigma and discrimination (due to HIV, race/ethnicity, and other intersectional identities), concepts interrelated with quality of health care, as they impact key health care interactions for Black and Latina women living with HIV.” | Five WIHS sites located in  Atlanta, GA, Birmingham, AL, Brooklyn, NY, Chapel Hill, NC, Chicago, IL, and Jackson, MS  Part of an exploratory mixed methods  sub-study to the Women’s Interagency HIV  Study (WIHS)  November 2017 - May 2018 | International Journal of Equity in Health | Mixed methods  Focus groups; in-depth interview  Two-stage inductive thematic analysis; initial framework of broad codes and subcodes were developed based on emerging patterns; NVivo 12 used for coding; compared coded transcripts to double-coded and revised; sub-team revised initial codebook; conducted secondary analysis to explore themes | 12 focus groups  (5-11 participants per group);  three individual in-depth interviews  Focus groups designed to foster intergroup discussion regarding stigma, discrimination, and healthcare interactions.  Interviews supplemented focus groups to engage women in the study who were participating in Spanish at locations with low  Spanish-speaking recruitment.  Semi-structured format to facilitate discussions about satisfaction and dissatisfaction with health care engagement experiences; suggestions for improvement | (n=92) WLWH | Themes emerged related to women’s health care satisfaction or dissatisfaction at the provider, clinic, and systems levels and across Institute of Medicine-defined quality of care domains (effectiveness, efficiency, equity, patient-centeredness, safety and timeliness). Women’s degree of care satisfaction was driven by: (1) knowledge-based care resulting in desired outcomes (effectiveness); (2) coordination, continuity and necessity of care (efficiency); (3) perceived disparities in care (equity); (4) care delivery characterized by compassion, nonjudgment, accommodation, and autonomous decision-making (patient-centeredness); (5) attention to avoiding side effects and over-medicalization (safety); and (6) limited wait time (timeliness). |
| 1. Robillard, A. et al. (2017) | Advice for prevention from HIV-positive African-American women: ‘My story is not just a story’ | “To document advice from HIV-positive African-American women to young African-American women, as described in their own cultural narratives collected through qualitative interviews.” | Two community-based organizations and one clinic serving PLWH in Columbia, South Carolina  September 2014 -  October 2015 | Culture, Health & Sexuality | Naturalistic qualitative design and grounded theory (Patton 2012).  Part of a larger mixed methods study  Semi-structured interviews and survey  Analyzed using NVivo;  portions capturing specific advice to young African American women were extracted; inductive analysis used to identify patterns; data coded into themes followed by confirmatory discussion | 1-2 hour interviews; average of 60 minutes  Used interview guide to expand on survey data and to explore older Black women’s experiences with HIV stigma, HIV care, and ART adherence.  Ecological approach used to capture the multiple levels operating in women’s lives: individual, interpersonal, community and institutional levels (McLeroy et al. 1988). Chronological approach was based on three distinct timeframes over the woman’s life course with respect to HIV: (1) before she contracted HIV, (2) during or around the time she thought she contracted HIV and (3) while living with HIV.  $50 incentive | (n=25) BWLWH  18+; African American; HIV positive | Themes: (1) advice for prevention, (2) support systems for prevention, (3) education, (4) empowerment/self-care and (5) potential barriers to prevention.  Advice reflected recommendations based on personal experience and SDOH linked to HIV, such as stigma, access to education and healthcare, social support, and gender and power dynamics  Communication with parents, family and friends regarding education and social support emerged as an important interpersonal factor for participants, as were interactions with sexual/romantic partners.  Stigma, at the community level, was consistently discussed as a hindrance to prevention. |
| 1. Sanders, L. (2008) | Women's Voices: the Lived Experience of Pregnancy and Motherhood after Diagnosis with HIV | “To explore the meaning  of pregnancy after diagnosis with HIV.” | Two academic health centers in metropolitan New York  June and August 2006 | Journal of the Association of Nurses in AIDS Care | Phenomenological methodology  Interviews  Giorgi’s (1985) method used to analyze the data; meaning units were formed and synthesized into a summary of the participants’ experiences and their meaning; consultants were used in this process to decrease the likelihood of researcher bias. | 45-90 minute interviews  Each participant was asked to describe her experience upon receiving the diagnosis of HIV; prompt such as, ‘‘*Tell me more*,’’ was used to encourage the participants to describe experience of pregnancy after diagnosis with HIV.  Provided a financial incentive of $50 | (n=9) WLWH  18+; fluent in English; had a diagnosis of HIV; currently pregnant or had a child after receiving a diagnosis of HIV. | Themes: (1) extreme emotional distress after HIV diagnosis; (2) feeling stigmatized; (3) emotions related to the pregnancy and the baby: women feared transmission of the infection to the baby, worried about the safety of ART, and were anxious about the effects of the pregnancy on their health; (4) experiences with health care providers: all participants experienced at least one encounter in which they reported feeling marginalized, vulnerable, and treated as less than a person related to having HIV; and (5) motherhood for women with a diagnosis of HIV: a means to rectify mistakes made with children born previously and to be a good, loving mother. |
| 1. Sangaramoorthy, T. et al. (2017) | HIV Stigma, Retention in Care, and Adherence Among Older Black Women  Living With HIV | “To explore HIV stigma, retention in care, and ART adherence in older Black women.” | Prince George’s County, MD  September 2014 - October 2015 | Journal of the Association of Nurses in AIDS Care | Modified grounded theory approach  (Charmaz 2014; Glaser and Strauss 1967)  Part of a larger mixed methods study  Interviews were analyzed using QSR NVivo 11; open codes used to identify themes; axial coding to refine; selected coding to group; final codebook included a mix 52 in vivo and descriptive codes, built around three major themes | 45-90 minute interviews; 60-minute average.  HIV stigma was measured using a 27-item stigma scale adapted from the Multidimensional Measure of Internalized HIV Stigma Scale.  Retention in care. Retention in care was measured using two indicators: (a) the number of HIV-related care visits scheduled in the past year and (b) number of kept visits of the total scheduled visits for HIV-related care.  ART adherence. ART adherence was measured using four questions: (a) *Do you ever forget to take your HIV* medication?; (b) *If you feel worse, do you stop taking your medication?;* (c) *Did you not take any of your HIV medications over the past weekend?*; and (d) *What is the total number of missed doses in the past 7 days?*  Participants were compensated $25. | (n=35) BWLWH  40+; Black or African American; had documentation of HIV seropositive status; had a primary residence in Prince George’s County, Maryland | Themes: (1) HIV stigma: *public silence.* HIV continued to be a stigmatized and stigmatizing subject in their communities. *Avoidance and rejection*. HIV stigma affected participants’ perceptions of disclosure. (2) experiences with retention in care and ART adherence: *Facilitators and barriers*: Participants used calendars, phone alarms, and reminder calls/cards to keep track of appointments or medication times. Barriers centered on structural challenges such as lack of access, coordination of care, and routine financial hardship. *Comorbidity:* Participants suffered from numerous comorbidities that required additional health services. (3) changes over the life-course: Participants were more accepting of the infection as they aged and were better able to cope with negative public attitudes and described a similar shift in their experiences with HIV care and ART adherence. |
| 1. Sangaramoorthy, T. et al. (2017) | Intersectional stigma among midlife and older Black women living with HIV | “Given the complex nature of intersectional stigma, we aim to contribute to the literature by examining how stigma manifests among midlife and older Black women living with HIV.” | Prince George’s County, MD  September 2014 - October 2015 | Culture, Health & Sexuality | Modified grounded theory approach  (Charmaz 2014; Glaser and Strauss 1967)  Part of a larger mixed methods study  Interviews were analyzed using QSR NVivo 11; open codes used to identify themes; axial coding to refine; selected coding to group; final codebook included a mix 52 in vivo and descriptive codes, built around three major themes | 45-90 minute interviews; 60-minute average.  Semi-structured interview guide developed from discussions with community partners, HIV-related stigma experts, and authors’ previous research.  Interview questions explored domains related to experiences of living with HIV, relationships with providers, facilitators and barriers to care, social relationships and support, and everyday experiences of gender, racial, and age discrimination; interviews also documented how perceptions and experiences related to these domains changed over time, from the moment of diagnosis to the present day.  Participants were compensated $25. | (n=35) BWLWH  40+; Black or African American; had documentation of HIV seropositive status; had a primary residence in Prince George’s County, Maryland | Themes: (1) manifestations of intersectional stigma: women’s narratives highlighted the inextricability of gender and race in discussions of HIV-related stigma.  (2) experiences of intersectional stigma: operated at multiple levels— from the interpersonal/familial to the broader community to institutional/structural contexts.  and (3) changes in stigma experiences over time: despite persistent experiences of stigma and discrimination, women reported that they were better able to cope with stigma and manage their condition as they aged. For many women, overcoming adversity and unfavorable life events, including HIV, increased their feelings of resiliency and competency. |
| 1. Scott A. (2009) | Illness Meanings of AIDS Among Women With HIV: Merging Immunology and Life Experience | Study question: “What does AIDS mean?”  No clear statement of aims. | HIV clinic in New Orleans, Louisiana, as part of a National Institute on Drug Abuse (NIDA) substudy  examining support women with HIV in an urban context | Qualitative Health Research | Qualitative descriptive with free listening and drawings  Analyzed using thematic analysis (Crabtree & Miller, 1999); segments with the word “AIDS” examined as a group; narrative summaries of each transcript; free listening examined quantitatively and qualitatively  ; drawings analyzed using Guillemin’s (2004) adaptation of Rose’s critical methodology (Rose, 2001). | 18 interviews total:  two rounds of in-depth interviews, 4 months apart  First interview focused on families, schools and work, life situations growing up,  public assistance, experiences at the clinic, with HAART, and with HIV.  In the second interview, participants were asked about AIDS and other HIV-related biomedical terms, including what they meant, how people used them in their communities and in the clinic, and how the terms made them feel.  Participants were compensated $30. | (n=10) BWLWH  Ages 18-24; African American or “mixed race” | Themes: (1) AIDS as death: most common word association to AIDS (2) distance from discredit: the meanings of AIDS held by the women incorporated not only physical collapse, but also moral and social collapse or discredit (3) importance of personal context: each woman negotiated HIV knowledge in the context of her own personal experience. (4) meanings of AIDS after an AIDS diagnosis: for the women in the study with AIDS diagnoses, the diagnosis was not simply a clinical event. It required a search for a personal illness meaning that could sustain hope in lieu of one that denoted their imminent death. |
| 1. Small et al. (2022) | Perceptions of healthcare accessibility and medical mistrust among Black women living with HIV in the USA | To explore the experiences of BWLWH in healthcare settings as they relate to HIV treatment accessibility and medical mistrust. | Los Angeles, CA  Two community-based centers  June - October 2019 | Culture, Health & Sexuality | Narrative and phenomenological approaches  Semi-structured focus groups  Thematic analysis used; open coding was conducted in Dedoose (version 8.3.44); analytical coding used to refine and combine codes; codebook was iteratively developed; codes were categorized by themes | 60 - 90 minutes focus groups  Four semi-structured focus groups, ranging from 4-8 women per group  Participants were asked about their experiences with healthcare providers, including prompts such as, *“What do you like about your healthcare provider?”* and *“What do you wish your healthcare provider would say to you during your visit?”* | (n=20) BWLWH  18+; identified as a Black woman; English speaking; prescribed and currently taking ART; and having the capacity to provide consent. | Used intersectionality and structural intersectionality as guiding frameworks.  Four themes were identified: (1) multilevel stigma and discrimination: the combination of HIV, race, class, and gender resulted in stigma, prejudice, and discrimination; (2) medical mistrust of providers across multiple settings; (3) varying responses to stigma, discrimination, and medical mistrust; and (4) preferences for patient-provider relationships: women desired to feel supported by health providers and staff. |
| 1. Subramaniam, S. et al. (2017) | Resilience in Low-income African American Women Living and Aging with HIV | “The goals of this study were twofold: (a) to gain an  understanding of how women dealt with and overcame health challenges, and (b) to identify factors that contribute to sustaining resilience.” | Metropolitan  area of a Midwestern state in the United States | Journal of Women & Aging | Grounded theory study using a constructivist framework (Charmaz, 2012).  Semi-structured  interview  Analyzed in NVivo 10; open coding used to identify initial codes; sorted and grouped into axial codes then into dominant themes | 30 minunte-2-hour interviews | (n=8) BWLWH  18+; self-identified with physical and/or mental health challenges over their lifetime | Resilience themes: (a) self-acceptance was a process; (b) optimism: encompassed being grateful, positive, having goals and aspirations; (c) support systems such as family and organizations; and (d) religion and spirituality: a means through which they drew strength, gave them hope.  Women showed resilience, despite having low incomes and enduring the stigma and secrecy. They were able to make the transformation from being a ‘sick’ person to incorporating the illness status into their identity. |
| 1. Teti, M. et al. (2015) | "I Created Something New with Something that Had Died": Photo-Narratives of Positive Transformation Among Women with HIV | “To uncover and understand  women’s text and visual examples of their positive life  transformations with HIV.” | AIDS service organizations in three U.S. cities in the Midwest and Northeast | AIDS Behavior | Photovoice methods  Focus groups; semi-structured individual interviews; exhibit  300 photographs, 15 group and 20 individual interviews; transcripts analyzed using Atlas.ti; Char- maz’ guidelines used for theme analysis; open coding followed by selective coding; generated coding reports; analytic notes used throughout | Three group photo-sharing and discussion sessions (4-8 participants), an individual interview with each participant, and a public photo exhibit.  After each participant presented and discussed her photos, the group discussed their reactions to the images.  Semi- structured guide included the following questions: *‘‘What does this photo capture about your life or story with HIV?’’ ‘‘What does the picture or issue mean to you?’’ ‘‘What challenges or strengths does the image convey?’’*  Following the exhibit, 1-2 hour individual interviews to explore experience in the project and give the women a chance to reflect on their pictures individually. | (n=30) WLWH | Four major transformations (1) transitions to health and wellness: common for participants to capture positive progression regarding their health; (2) commitment to spirituality: women became stronger in their faith or spirituality as part of their illness experience; (3) embracing self-acceptance: women transitioned to accept various aspects of HIV and their lives with HIV; and (4) becoming more confident: often the most important thing that they learned about themselves through living with HIV. |
| 1. Tufts, K. et al. (2010) | Self-Care Behaviors of African American Women Living with HIV: A Qualitative Perspective | “To systematically collect data about the SC experiences of these women.” | HIV education, resources, and consultation center in a Southeastern metropolitan city in the United States | Journal of the Association of Nurses in AIDS Care | Qualitative descriptive  Data were analyzed using content analysis (Coffey & Atkinson, 1996).  Investigators individually conducted line-by-line coding, developing categories and core concepts; compared results and discussed discrepancies; established a consolidated coding scheme; synthesized into themes | 90-135 minute focus groups  Five focus groups  Structured discussion guide used  Conducted in private conference room  Compensated with $25 grocery store gift card. | (n=21) BWLWH  Ages 18-65; self-identified as AA; had been diagnosed with HIV; resided in a metropolitan area in the southeastern region of the United States | Themes: (1) me first: putting one’s health and well-being before the health and well-being of others, calling it self-preference; and (2) mind, body, and spirit: taking care of one’s self emotionally, mentally, or spiritually; pampering as a sub-theme  Self-care behaviors: (1) do what the doctor says: maintaining medical regimens that consisted of seeking the care and advice of a health care team; and (2) living healthy: (a) prayer, singing, and church, (b) surround myself with supportive people, (c) maintaining recovery, and (d) I can tell.  Facilitators of self-care: spirituality, social support and acceptance  Barriers to self-care: caregiving, negative attitudes, stigma and nondisclosure |
| 1. Watkins-Hayes, C. et al. (2012) | ‘Dying From’ To ‘Living with’: Framing Institutions and the Coping Processes  of African American women Living with HIV/AIDS | “To explicate the effects of HIV on four social domains: social support, labor force participation, child bearing and rearing, and intimate relationships.” | Chicago, IL  2005 - 2008 | Social Science & Medicine | Grounded theory  Transcripts coded in two stages using HyperResearch; first coded following themes identified in interview guide; recoded using a new set of emergent codes; concept of the framing institution emerged | Two in-depth semi-structured interviews with thirty HIV-positive women | (n=30) BWLWH  Ages 18-55 | Framing institutions (1) help women address health crises by providing medical information and care or drug and alcohol treatment; (2) encourage women to finally accept being HIV-positive by providing a language, shared knowledge, and a support structure in a destigmatizing setting; and (3) connect women with resources that support the long-term coping process.  Coping is an ongoing process. Feelings of internal shame and external stigma threaten to throw them “off course” at any time.  Those who do not experience extreme economic marginalization because of their class status still grapple with intersectional stigma on the basis of their racial, gender, and HIV statuses. |
| 1. Williams, R. et al. (2021) | The Meaning and Perceptions of HIV-Related  Stigma in African American Women Living With HIV  in Rural Florida: A Qualitative Study | “To explore the meaning and perceptions of HIV-related stigma among a sample of African American women living with HIV in Florida.” | Rural Florida  May 2018 - March 2019  Data were drawn from a 30-day pilot study of the Wisepill medication event monitoring system (MEMS) | Journal of the Association of Nurses in AIDS Care | phenomenological approach  Semi-structured interviews  NVIVO 12 Plus; independently clustered similar or repetitive responses and iteratively reviewed for consensus; Participant perspectives were consolidated into second-order observer constructs; codebook developed and maintained | Interview questions informed by the HIV-related Stigma, Engagement in Care, and Health Outcomes conceptual framework  Began the interview by exploring their personal meaning of HIV-related stigma. Subsequent questions assessed perceptions of HIV stigma in healthcare and stigma in the context of co-occurring illnesses  Compensated up to $75 | (n=13) WLWH  18+; African American women; self-reported an HIV diagnosis; prescribed antiretroviral medications; and not a student or employee at the University of Florida | Observer perspectives included: (a) self-conceptualization: beliefs and attitudes formed in response to HIV and stigma; (b) experiences, such as judgment, stereotypes, prejudice, and discrimination of PLWH (c) intersectionality:  the interconnectedness of marginalized identity(s) and its effects on living with HIV (d) psychological dysfunction: how the social environment affects mental/physical health and overall quality of life, and (e) overcoming stigma: adaptive and maladaptive ways individuals manage living with HIV |
