# Supplementary material for: A qualitative metasynthesis of stigma in women living with HIV in the United States
Source: Int J Equity Health. 2023 Aug 21;22:158. doi: 10.1186/s12939-023-01969-5 (PMC10441719; doi:10.1186/s12939-023-01969-5)
Supplement: Supplementary file 2 — Supplementary Material 2 [file 12939_2023_1969_MOESM2_ESM.docx]

| **Table S2.** Quality Assessment | | | | | | | | | | | |
| --- | --- | --- | --- | --- | --- | --- | --- | --- | --- | --- | --- |
|  | Item 1. Was there a clear statement of the aims of the research? | Item 2. Is a qualitative methodology appropriate? | Item 3. Was the research design appropriate to address the aims of the research? | Item 4. Was the recruitment strategy appropriate to the aims of the research? | Item 5. Was the data collected in a way that addressed the research issue? | Item 6. Has the relationship between researcher and participants been adequately considered? | Item 7. Have ethical issues been taken into consideration? | Item 8. Was the data analysis sufficiently rigorous? | Item 9. Is there a clear statement of findings? | Item 10. Was this research valuable? | Overall score |
| 1. Buchberg, M. et al. (2015) | Y | Y | Y | Y | Y | N | Y | Y | Y | Y | 9 |
| 1. Buseh, A. et al. (2006) | Y | Y | Y | Y | Y | U | Y | Y | Y | Y | 9.5 |
| 1. Caiola, C. et al. (2017) | Y | Y | Y | Y | Y | Y | Y | Y | Y | Y | 10 |
| 1. Carr, R. & Gramling, L. (2004) | Y | Y | Y | Y | Y | N | Y | Y | N | Y | 8 |
| 1. Cuca, Y. & Rose, C. (2016) | Y | Y | Y | Y | Y | N | Y | Y | Y | Y | 9 |
| 1. Dale, S. et al. (2018) | Y | Y | Y | Y | Y | Y | Y | Y | Y | Y | 10 |
| 1. Dale, S. & Safren, S. (2018) | Y | Y | Y | Y | Y | Y | Y | Y | Y | Y | 10 |
| 1. Davis, K. et al. (2021) | Y | Y | Y | Y | Y | U | Y | Y | Y | Y | 9.5 |
| 1. Davtyan, M. et al. (2016) | Y | Y | Y | Y | Y | U | Y | Y | Y | Y | 9.5 |
| 1. Fair, C. & Brackett, B. (2008) | Y | Y | Y | Y | Y | N | Y | Y | Y | Y | 9 |
| 1. Fernandez, S. et al. (2022) | Y | Y | Y | Y | Y | Y | Y | Y | Y | Y | 10 |
| 1. Fletcher, F. et al. (2016) | Y | Y | Y | Y | Y | N | Y | Y | Y | Y | 9 |
| 1. Fletcher, F. et al. (2020) | Y | Y | Y | Y | Y | Y | Y | Y | Y | Y | 10 |
| 1. Grodensky, C. et al. (2015) | Y | Y | Y | Y | Y | N | Y | Y | Y | Y | 9 |
| 1. Hampton, C. & Gillum, T. (2020) | Y | Y | Y | Y | Y | U | Y | Y | Y | Y | 9.5 |
| 1. James-Borga, J. & Frederickson, K. (2018) | Y | Y | Y | Y | Y | Y | Y | Y | Y | Y | 10 |
| 1. Kempf, M. et al. (2010) | Y | Y | Y | Y | Y | U | Y | Y | Y | Y | 9.5 |
| 1. Kim, S.-J. et al. (2021) | Y | Y | Y | Y | Y | U | Y | Y | Y | Y | 9.5 |
| 1. Koch, A. et al. (2022) | Y | Y | Y | Y | Y | Y | Y | Y | Y | Y | 10 |
| 1. Lekas, H-M et al. (2006) | Y | Y | Y | Y | Y | Y | Y | Y | Y | Y | 10 |
| 1. Marg, L. et al. (2020) | Y | Y | Y | Y | Y | Y | Y | Y | Y | Y | 10 |
| 1. McDoom, M. et al. (2015) | Y | Y | Y | Y | Y | Y | Y | Y | Y | Y | 10 |
| 1. McMillian-Bohler, J. et al. (2023) | Y | Y | Y | Y | Y | Y | Y | Y | Y | Y | 10 |
| 1. Ojukwu, E. et al. (2022) | Y | Y | Y | Y | Y | Y | Y | Y | Y | Y | 10 |
| 1. Peltzer, J. et. al. (2015) | Y | Y | Y | Y | Y | N | Y | Y | Y | Y | 9 |
| 1. Peltzer, J. et al. (2016). | Y | Y | Y | Y | Y | N | Y | Y | Y | Y | 9 |
| 1. Phillips, K. et al. (2011) | Y | Y | Y | Y | Y | N/A | Y | Y | Y | Y | 10 |
| 1. Qiao, S. et al. (2021) | Y | Y | Y | Y | Y | N | Y | Y | Y | Y | 9 |
| 1. Relf, M. et al. (2015) | Y | Y | Y | Y | Y | N/A | Y | Y | Y | Y | 10 |
| 1. Rice, W. et al. (2018) | Y | Y | Y | Y | Y | Y | Y | Y | Y | Y | 10 |
| 1. Rice, W. et al. (2019) | Y | Y | Y | Y | Y | Y | Y | Y | Y | Y | 10 |
| 1. Rice, W. et al. (2020) | Y | Y | Y | Y | Y | Y | Y | Y | Y | Y | 10 |
| 1. Robillard, A. et al. (2017) | Y | Y | Y | Y | Y | Y | Y | Y | Y | Y | 10 |
| 1. Sanders, L. (2008) | Y | Y | Y | Y | Y | U | Y | Y | Y | Y | 9.5 |
| 1. Sangaramoorthy, T. et al. (2017) | Y | Y | Y | Y | Y | N | Y | Y | Y | Y | 9 |
| 1. Sangaramoorthy, T. et al. (2017) | Y | Y | Y | Y | Y | U | Y | Y | Y | Y | 9.5 |
| 1. Scott A. (2009) | Y | Y | Y | Y | Y | N | Y | Y | Y | Y | 9 |
| 1. Small, L. et al. (2022) | Y | Y | Y | Y | Y | Y | Y | Y | Y | Y | 10 |
| 1. Subramaniam, S. et al. (2017) | Y | Y | Y | Y | Y | Y | Y | Y | Y | Y | 10 |
| 1. Teti, M. et al. (2015) | Y | Y | Y | Y | Y | U | Y | Y | Y | Y | 9.5 |
| 1. Tufts, K. et al. (2010) | Y | Y | Y | Y | Y | Y | Y | Y | Y | Y | 10 |
| 1. Watkins-Hayes, C. et al. (2012) | Y | Y | Y | Y | Y | N | Y | Y | Y | Y | 9 |
| 1. Williams, R. et al. (2021) | Y | Y | Y | Y | Y | Y | Y | Y | Y | Y | 10 |

Y = Yes (1); N = No (0); U = Unclear (0.5)

Critical Appraisal Skills Programme (CASP) Qualitative Checklist
